# Early Gut Microbiota and Neurodevelopmental Trajectories: Implications for Pediatric Neuropsychiatric Vulnerability—A Narrative Review

**DOI:** 10.3390/nu18101541

**Published:** 2026-05-13

**Authors:** Vasile Valeriu Lupu, Alin Horatiu Nedelcu, Ingrith Miron, Sorana Caterina Anton, Maria Oana Sasaran, Otilia Elena Frasinariu, Elena Jechel, Laura Iulia Bozomitu, Tatiana Chisnoiu, Carmen Rodica Anton, Cristina Oana Marginean, Ionela Daniela Morariu, Cristina Maria Mihai, Emil Anton, Ancuta Lupu

**Affiliations:** 1 Grigore T. Popa University of Medicine and Pharmacy, 700115 Iasi, Romania; vasile.lupu@umfiasi.ro (V.V.L.); alin.nedelcu@umfiasi.ro (A.H.N.); ingrith.miron@umfiasi.ro (I.M.); sorana.anton@umfiasi.ro (S.C.A.); frasinariu.otilia@umfiasi.ro (O.E.F.); laura.bozomitu@umfiasi.ro (L.I.B.); carmen.anton@umfiasi.ro (C.R.A.); ionela.morariu@umfiasi.ro (I.D.M.); emil.anton@umfiasi.ro (E.A.); ancuta.ignat1@umfiasi.ro (A.L.); 2 Department of Pediatrics, “George Emil Palade” University of Medicine, Pharmacy, Science and Technology, 540142 Targu Mures, Romania; oanam93@yahoo.com (M.O.S.); marginean.oana@gmail.com (C.O.M.); 3 Department of Pediatrics, Faculty of Medicine, “Ovidius” University, 900470 Constanta, Romania; tatiana.chisnoiu@365.univ-ovidius.ro (T.C.); cristina_mihai@365.univ-ovidius.ro (C.M.M.)

**Keywords:** gut microbiota, gut–brain axis, neurodevelopment, early life programming, nutrition, pediatric neuropsychiatric disorders

## Abstract

Neurodevelopment is a dynamic and multifactorial process, critical in the early stages of life, involving the formation of neural networks, the establishment of synapses, and the maturation of cognitive, social and emotional circuits. In this context, the gut microbiome emerges as an essential regulator of neurodevelopment, exerting influences through multiple biochemical and immunological mechanisms that define the “gut-brain axis”. The microbiota modulates neurodevelopment by regulating neurotransmitters (serotonin, dopamine, GABA), the production of microbial metabolites, including short-chain fatty acids, the modulation of inflammatory cytokines, and vagal signaling to the central nervous system. Recent evidence highlights the role of microbiota in modulating microglia, synaptogenesis, dendritic maturation, and neuronal plasticity, emphasizing how these processes are influenced by microbial activity rather than providing a comprehensive treatise on plasticity itself. Gut microbiota disturbances, or dysbiosis, have been associated with various neuropsychiatric and neurodevelopmental disorders, contributing to cognitive, behavioral, and emotional dysfunctions. This article summarizes, in a narrative manner, the main dysbiosis patterns identified in these disorders and the biological mechanisms by which the microbiome influences neuronal development and function, including immune–neuronal interactions, metabolomic modulation, and neuroendocrine signaling. Finally, emerging directions of intervention aimed at adjusting the microbial profile, such as dietary adjustment, the use of probiotics, prebiotics, symbiotics, and fecal microbiota transplantation, are presented with the aim of positively influencing neurodevelopment and preventing or ameliorating associated dysfunctions. This review emphasizes the need for longitudinal, rigorous, and controlled clinical trials to validate the efficacy of microbiota modulation strategies and to substantiate their integration into individualized pediatric management protocols.

## 1. Introduction

Neurological development in the infant is a dynamic and multifactorial process, starting in the intrauterine period and continuing intensively in the first years of life. The first weeks of gestation are marked mainly by neurogenesis, a process leading to the formation of neurons and glial cells, and neuronal migration, responsible for the establishment of cortical and subcortical architecture. In the second trimester, synaptogenesis and dendritic arborization progress rapidly, and axons begin to myelinate, facilitating the efficient transmission of nerve signals [1,2,3]. After birth, plasticity remains extremely pronounced, with synapses being shaped by sensory experiences, cognitive stimulation, and social interactions. Optimal development in childhood is essential for long-term neurological health, as it strengthens the foundations of cognitive, emotional, and social functioning, influencing educational performance and the risk of neuropsychiatric disorders in adulthood [4,5]. The factors involved in the modulation of neurodevelopment are multiple, including prenatal and postnatal aspects. Prenatal factors can include genetics, epigenetics, maternal nutrition (especially folic acid intake, omega-3 fatty acids, vitamins B and D), exposure to toxins (alcohol, nicotine, heavy metals), intrauterine infections, and maternal stress, which can activate inflammatory responses and affect the fetal hypothalamic–pituitary–adrenocortical axis [3,6]. Postnatally, infant nutrition, sleep quality, social interaction, cognitive stimulation, as well as the intestinal microbial environment, influence neurological development [7].

Focusing our attention on the gut microbiome, we note the particular impact it plays on immune system development, metabolism, intestinal barrier integrity, and inflammatory response, with major implications in determining susceptibility to multiple pathologies. Imbalances in the gut microbiota during critical periods of development have been associated with a wide range of pediatric conditions, including allergic, metabolic, gastrointestinal, and neuropsychiatric diseases, highlighting its importance as a potential determinant of long-term health [8,9,10]. Through the gut–brain axis, this microbial community influences the development and function of the central nervous system (CNS), modulating neurotransmitters (e.g., serotonin, dopamine, and gamma-aminobutyric acid—GABA), producing biologically active metabolites (e.g., short-chain fatty acids—SCFA’s), and influencing the systemic immune response. These serve as signals and they affect synaptic plasticity, the development of neural circuits, and the regulation of the stress response, thereby impacting cognition, behavior, and emotional regulation. Disturbances in the microbiota or deficiencies in other critical postnatal factors can generate cognitive and behavioral vulnerabilities and increase the risk of neuropsychiatric disorders, highlighting the importance of optimal neurological development in the first years of life [11,12].

In the context of growing interest in the role of the gut microbiome in pediatric neuropsychiatric health, this article presents a structured narrative review of the recent literature, aiming to synthesize and critically discuss the available evidence regarding the influence of microbiota on neurocognitive and behavioral development in children. A literature search was conducted using major scientific databases, including PubMed, Scopus, Web of Science, and Embase, focusing on articles published within the last 10 years. The search strategy included combinations of relevant keywords such as “gut microbiota”, “gut–brain axis”, “neurodevelopment”, “early life microbiome”, “microglia”, “short-chain fatty acids”, “pediatric neuropsychiatric disorders”, and “early life programming”. Studies were selected based on their relevance to the scope of the review, with emphasis on those directly addressing the relationship between gut microbiota and neurodevelopmental or behavioral outcomes, particularly in early-life contexts. Greater consideration was given to studies with clearer methodological reporting and stronger designs, including longitudinal human studies, controlled experimental models, systematic reviews, and meta-analyses, while studies with limited methodological detail or indirect relevance were interpreted with appropriate caution. Both preclinical (animal-based) and clinical (human) studies were included and analyzed separately to ensure a clear distinction between evidence levels and to appropriately reflect their respective translational relevance, thereby minimizing the risk of overinterpretation or bias. It should be emphasized that this structured narrative review aims to provide a focused synthesis rather than an exhaustive or systematic coverage of the literature. At the same time, the review adopts a critical interpretative framework, explicitly highlighting inconsistencies, methodological variability, and conflicting findings across studies, rather than aiming for comprehensive inclusion of all available evidence. Accordingly, the objective is to critically discuss how early-life gut microbiota may influence synaptic plasticity, neurotransmitter receptor dynamics, and related neurodevelopmental processes, while maintaining a clear separation between preclinical and clinical evidence in order to appropriately reflect translational relevance and avoid overinterpretation of associative findings. This approach allows methodological transparency while preserving the flexibility required to synthesize heterogeneous evidence across studies, without implying a systematic review methodology.

## 2. The Gut Microbiome and the Gut–Brain Axis

The gut microbiome is the complex community of microorganisms that colonize the gastrointestinal tract, being essential for maintaining a functional microbial balance. The diversity and stability of this community are critical factors for the resilience of the ecosystem, influencing not only digestive processes, but also energy metabolism, modulation of immune responses, and metabolism of exogenous substances (xenobiotics). The microbiome contributes to the synthesis of vitamins and digestive enzyme cofactors, the metabolism of dietary fibers, and the production of metabolites with an anti-inflammatory role, which support intestinal function and systemic homeostasis. The microbiome can also modulate hormonal secretion, having an indirect impact on the regulation of blood glucose and lipid metabolism [13,14,15,16]. The composition of the microbiota exhibits the greatest variability during the first 12 months of life, remaining in a perpetual state of change until approximately 3–5 years of age. Disruptions of this microbial community during critical periods of development have been associated with an increased risk of metabolic, allergic, and inflammatory diseases, highlighting the importance of maintaining a balanced microbiota from the early years of life. This perspective highlights the microbiome not only as a local ecosystem, but as a regulator of systemic health and the body’s adaptation to environmental and dietary factors over the long term [17,18,19].

The gut–brain axis represents a bidirectional network of functional communication between the gastrointestinal tract and the CNS, which can explain how gut health can influence general well-being, behavior and cognitive functions, and vice versa. Thus, disturbances in the gut—e.g., digestive disorders, chronic inflammation, or microbiota imbalance—can be associated with changes in mood, energy levels, emotional regulation, and concentration. Similarly, psychological or environmental factors, such as stress, anxiety, or sleep deprivation, can affect gut function, influencing digestion, motility, and gastrointestinal comfort [20,21,22,23]. Thus, the gut–brain axis emphasizes the interdependence between the gut, cognitive functions, and behavior, constituting an essential framework for understanding pediatric health, gastrointestinal, and neuropsychiatric disorders. Bidirectional communication between the gut and the brain is achieved through a complex network of neuronal, hormonal, immunological, and metabolic signaling, each pathway contributing to the fine-tuning of physiological and cognitive functions. This perspective supports integrated prevention and intervention strategies, including optimizing diet, lifestyle, and psychosocial support, designed to support healthy development and well-being in children [24,25,26].

The neural pathway of the gut–brain axis is mediated mainly by the vagus nerve and the enteric nervous system (ENS), representing one of the fastest and most specific pathways for transmitting intestinal signals to the CNS [27,28]. Vagal afferent fibers detect changes in intestinal distension, luminal chemical composition, and microbial metabolic activity, including signals from bacterial metabolites, transmitting this information to the nucleus of the solitary tract and subsequently to limbic circuits involved in the regulation of stress, eating behavior, and emotional processing [28,29]. The ENS, often referred to as the “second brain”, represents an extensive neuronal network organized in the myoenteric and submucosal plexuses, capable of autonomic reflex activity, but also of bidirectional interaction with the CNS [30]. The ENS acts as a local integrator of mechanical, chemical, and microbial signals, coordinating gastrointestinal motility, digestive secretions, and blood flow, while enteric neurons and glial cells respond to microbial metabolites and inflammatory mediators, contributing to the maintenance of intestinal homeostasis [30,31]. The integrity and functional maturation of this neural pathway are essential for establishing effective gut–brain communication during development. Early disruptions in vagal signaling or ENS function may alter central processing of peripheral signals and contribute to persistent neurobehavioral vulnerabilities [28,29].

The endocrine pathway of the gut–brain axis is mediated by enteroendocrine cells, specialized epithelium capable of detecting nutritional and microbial stimuli in the lumen and transforming them into systemic hormonal signals [28,32]. These cells express G protein-coupled receptors and other molecular sensors, including Free Fatty Acid Receptors and Takeda G-protein coupled receptor 5, that are sensitive to nutrients, microbial metabolites, and fermentation products such as SCFA’s, bile acids, and tryptophan, thereby integrating signals regarding diet, host metabolic state, and gut microbiota activity [32,33]. In response to these signals, enteroendocrine cells secrete gastrointestinal hormones, such as GLP-1, PYY, cholecystokinin, and ghrelin, with paracrine, endocrine, and neurocrine effects, which transmit information to the CNS through both the systemic circulation, influencing hypothalamic and cortical structures involved in energy regulation, and by activating vagal afferent fibers [28,34]. Gut hormonal signaling regulates appetite, eating behavior, gastrointestinal motility, and energy balance, adapting physiological responses to microbial and nutritional changes. In parallel, enteroendocrine hormones interact with the hypothalamic–pituitary–adrenal axis, modulating the neuroendocrine response to stress and facilitating the body’s adaptation to internal and external factors [33,35]. Through these mechanisms, the endocrine pathway integrates peripheral, nutritional, and metabolic signals with central circuits, promoting both systemic homeostasis and the functional maturation of neuroendocrine networks [28,32].

Immune signaling constitutes a major communication pathway between the gut and the CNS, integrating information about the composition of the microbiota, the integrity of the intestinal barrier, and the metabolic state of the host [28,36]. Intestinal immune cells, including macrophages, dendritic cells, and T and B lymphocytes, secrete a wide range of soluble mediators—pro- and anti-inflammatory cytokines, chemokines, and microbial metabolites with immunomodulatory effect [37,38]. These mediators can influence the CNS through indirect pathways, including activation of vagal afferent fibers to the nucleus of the solitary tract and signaling through endothelial cells of the blood–brain barrier (BBB), modulating the phenotypes of microglia and astrocytes, as well as their capacity for synaptic remodeling and excitatory–inhibitory regulation of neuronal circuits [29,37,39]. Intestinal immune signaling regulates neuronal plasticity, synaptogenesis, and excitatory–inhibitory balance of neuronal circuits, contributing to cognitive and behavioral development [37,39]. During critical periods of development, intestinal dysbiosis, persistent systemic inflammation, or disproportionate activation of immune pathways can disrupt this communication, affecting microglial maturation, synaptic formation, and response to emotional and stress stimuli [36,38].

Also, microbial metabolites, such as SCFA’s (acetate, propionate, butyrate), neurotransmitters of microbial origin (GABA, serotonin, dopamine), and derived amino acids, play a central role in modulating gut–brain interactions [28,36,38]. At the local level, these mediators regulate the function of the intestinal epithelium by modulating the expression of tight junction proteins and mucus secretion, as well as the activity of enteric neurons, thus contributing to the control of motility and digestive secretions [28,40]. At the systemic level, microbial metabolites enter the circulation and coordinate peripheral immune cells, activating or inhibiting pro- and anti-inflammatory pathways and regulating the secretion of cytokines and chemokines [38,41]. In the CNS, these metabolites can directly or indirectly influence synaptic plasticity, microglial maturation, and the regulation of neuronal excitability, via endocrine, neuronal, and immunological signals [36,41]. The integrity of the intestinal barrier is essential in this process, with increased permeability allowing pro-inflammatory metabolites and microbial components to enter the circulation, with potentially neurotoxic or neuroinflammatory effects [40,41]. During critical periods of development, these interactions influence neural circuit formation, stress response, and cognitive-behavioral maturation, highlighting the fundamental role of the microbiome in regulating homeostasis and neuro-immune health [28,36,38].

The neonatal period and the first years of life constitute a critical window of biological plasticity, in which the processes of neuronal maturation, immune development, and intestinal colonization occur concurrently and interdependently. This synchronization allows for a functional balance between neurons, glial cells, immunocompetent cells, and the intestinal microbial ecosystem, which is essential for the formation and optimal organization of synaptic circuits, the regulation of the adaptive immune response, and the maintenance of the gut–brain barrier integrity [42,43]. Early colonization with beneficial microbes, together with the maturation of neural networks and the immune compartment, generates synergistic molecular signals that are self-sustaining, promoting optimal development and strengthening neuro-metabolic and neuro-endocrine functions [44,45].

**Figure 1:**
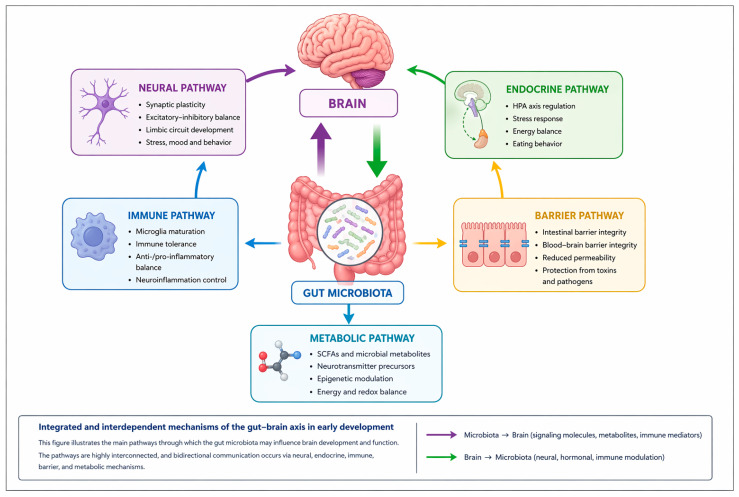
The neuro-immuno-microbial network in early development. The diagram illustrates the network of bidirectional interactions between the gut and the central nervous system, mediated by neuronal (vagus nerve and enteric nervous system), endocrine (enteroendocrine hormones), and immune (cytokines and immune cells) pathways. Microbial metabolites (e.g., short-chain fatty acids and neurotransmitters) contribute to the regulation of intestinal barrier function, immune responses, and neuronal activity. The figure highlights the integrated and interdependent nature of these mechanisms, emphasizing their coordinated function across systems. Further details are provided in the main text (Section 2 and Section 3).

Disturbances in the gut microbiota (dysbiosis), broadly defined as a persistent alteration in the composition, diversity, and functional capacity of the microbial ecosystem associated with a loss of host–microbiota homeostasis, may be particularly impactful during early-life critical developmental windows, when the coordination of neuronal and glial maturation, synaptic plasticity, myelination, and BBB integrity is especially vulnerable. These alterations increase vulnerability to adverse neurocognitive and behavioral outcomes, including anxiety- and depression-related behaviors, attentional deficits, impaired cognitive function, and behavioral abnormalities [12,45]. Genetic, maternal (intrauterine stress, inadequate diet, inflammation or drug exposure), and environmental (prematurity, nutrition, antibiotics) factors can amplify the effects of dysbiosis, highlighting the importance of maintaining a balanced intestinal ecosystem in the early period. Interventions that target microbiota balance—through dietary, prebiotic or probiotic strategies—can support neurological and immune development, reducing the risk of neuropsychiatric dysfunction and promoting an optimal cognitive and behavioral profile in the long term [36,46,47]. Figure 1 illustrates the parallel and intermodulated development of the three systems, integrating the information presented here with that discussed previously.

## 3. Pathophysiological Implications in Neurodevelopment

In this chapter, we examine how the gut microbiome may be involved in the development and function of the central nervous system. Given the multifactorial nature of pediatric neurodevelopmental disorders—including autism spectrum disorder (ASD), attention deficit hyperactivity disorder (ADHD), depression, and others—this chapter provides a broad overview while focusing on mechanistic pathways that have been proposed to be influenced by the gut microbiome. Much of the available evidence is derived from preclinical and in vitro studies, and should therefore be interpreted as suggestive rather than directly confirmatory in humans. The microbiota produces a range of bioactive metabolites, including SCFAs—acetate, propionate, and butyrate—as well as neurotransmitters or their precursors (GABA, serotonin, dopamine). These compounds are thought to function as signaling molecules and have been associated in experimental models with modulation of microglial activity, excitatory–inhibitory balance, synaptic plasticity, and inflammatory and hormonal responses during early brain development. At the molecular level, SCFAs have been shown in preclinical systems to influence gene expression through epigenetic mechanisms, particularly via inhibition of histone deacetylases (HDACs), leading to chromatin remodeling and transcriptional changes. Experimental studies have reported associations with genes involved in synaptic plasticity and neuronal metabolism, including BDNF and PGC-1α, as well as glial and immune-related genes such as GFAP, AHR, and cytokines IL-1β, IL-6, TNF-α, and IL-10. These effects are proposed to be mediated through intracellular signaling pathways (NF-κB, MAPK, STAT3) and antioxidant pathways (Nrf2/HO-1), which have been implicated in the regulation of neuroinflammatory responses, synaptogenesis, dendritic maturation, and synaptic receptor composition in preclinical models. Through these mechanisms, SCFAs have been suggested to contribute to dendritic spine formation and stabilization, as well as to influence the density and functional properties of glutamatergic receptors (NMDA, AMPA), potentially affecting excitatory synaptic transmission and plasticity. Microbial neurotransmitters and metabolites may further support excitatory–inhibitory balance and neuronal synchrony, and together with hormonal and immune signaling, are hypothesized to participate in the coordination of cortical and limbic circuit development. These processes may also involve modulation of the hypothalamic–pituitary–adrenal (HPA) axis, which regulates stress responses and may influence neural network maturation through both signaling pathways and epigenetic regulation of glucocorticoid-responsive genes in limbic and hypothalamic regions. Although these findings provide a coherent mechanistic framework, it should be emphasized that they are largely based on preclinical evidence, and translation to human neurodevelopment remains incomplete. Importantly, there is currently no direct evidence linking specific genes or pathways described above to gut microbiome alterations in human neurodevelopmental disorders such as ADHD or ASD. While preclinical findings are informative for hypothesis generation, human data remain heterogeneous. For instance, alterations in Bacteroides have been more consistently reported in ASD, whereas findings in ADHD are more variable and sometimes contradictory across studies. Overall, the relative contribution of microbiome-derived metabolites compared with host genetic, metabolic, and environmental factors remains unresolved, highlighting the complexity and multifactorial nature of these interactions [48,49,50].

### 3.1. Impact on Neuronal Development and the Role of Microglia

Microglia represent the principal immune cell population of the central nervous system (CNS), and play important roles in neuronal development and synaptic plasticity, in addition to their established function in maintaining tissue homeostasis through pathogen sensing and clearance of cellular debris. These multifunctional cells are involved in the regulation of neurogenesis, synaptic remodeling, and central inflammatory responses, and are considered important contributors to the maturation of neuronal circuits and the maintenance of excitatory–inhibitory balance in the developing cortex [51,52,53]. Microglial activity has been proposed to be influenced by metabolites and signaling molecules derived from the gut microbiota. Experimental and preclinical evidence suggests that such microbial-derived signals may interact with microglia through metabolic and immune-related pathways, potentially affecting their activation states, phagocytic activity, and cytokine production profiles. These interactions have been hypothesized to contribute to processes such as neuronal maturation, synaptogenesis, and neural network plasticity; however, direct causal evidence in humans remains limited, and current understanding is largely based on experimental models [49,54,55].

Alterations in microglial function, which may arise in association with microbial dysbiosis, genetic susceptibility, or environmental exposures, have been linked in experimental and observational studies to changes in myelination, blood–brain barrier integrity, and synaptic pruning efficiency. Such alterations have been associated with increased vulnerability to neurodevelopmental and neuropsychiatric conditions, including autism spectrum disorder, depression, ADHD, and other cognitive or behavioral disturbances; however, these associations remain complex and not yet mechanistically established in humans [51,52,56]. In addition, microglia are key mediators of the CNS response to injury, infection, and systemic inflammation. Their potential modulation by microbiota-derived signals has led to the hypothesis that the gut microbiome may contribute to CNS homeostasis, particularly during early developmental periods when neurogenesis and synaptic circuit formation are highly sensitive to internal and external environmental influences [49,54].

### 3.2. Synapse Formation and Neuronal Plasticity

Synaptic formation and neuronal plasticity are fundamental processes involved in the organization and refinement of neuronal circuits, contributing to cognitive functions and behavioral regulation [57,58]. Synaptogenesis involves dendritic arbor growth, development of presynaptic release sites, postsynaptic receptor localization, coordinated neuron–glia interactions, neurotrophin-mediated signaling (BDNF, NGF), and transcriptional regulation, which together are thought to guide circuit connectivity during development [57,59]. Synaptic plasticity, reflected by changes in transmission efficiency, receptor distribution, and dendritic spine remodeling, is considered to enable neural networks to adapt dynamically to experience [57,58]. Extracellular metabolic, immunological, and hormonal signals have been proposed to modulate these processes, potentially contributing to the integration of environmental inputs into circuit maturation; however, much of this evidence remains indirect or derived from experimental models [60,61].

SCFAs have been shown in preclinical studies to act as epigenetic modulators, potentially influencing DNA methylation and histone acetylation processes. These effects have been associated in experimental systems with regulation of genes involved in dendritic spine formation and stabilization, as well as with modulation of glutamatergic receptor composition, including NMDA and AMPA receptors, thereby potentially affecting excitatory synaptic properties [55,60]. Microbial neurotransmitters have been suggested to influence neuronal excitability in experimental models and may contribute to the regulation of excitatory–inhibitory balance, with potential implications for synchronization of neural network activity; however, direct evidence in human neurodevelopment remains limited [60,61]. In addition, microglia and astrocytes have been shown in preclinical models to respond to microbial-derived signals and participate in synaptic pruning processes, thereby contributing to the selective refinement of neural circuits during development. These mechanisms have been proposed to support experience-dependent plasticity and circuit maturation, although their translational relevance to human neurodevelopment is still under investigation [55,61]. Disruptions in these processes—potentially associated with gut dysbiosis, systemic inflammation, oxidative stress, or genetic vulnerability—have been linked in observational and experimental studies to alterations in dendritic spine stability, receptor composition, myelination, and neuronal synchronization, and have been associated with increased susceptibility to cognitive, behavioral, and emotional disorders [60,61]. However, these associations remain complex and do not establish causal relationships in humans. Overall, this body of evidence supports a proposed involvement of the gut–brain axis in shaping neurodevelopmental processes and neural circuit maturation. While disorder-specific differences exist, several overlapping mechanisms—including SCFA-related epigenetic modulation, neurotransmitter signaling, and microglial activity—have been suggested in preclinical literature as potentially converging pathways; however, their integration into a unified causal framework remains speculative at the current stage of evidence.

### 3.3. Influence on the Maturation of the Hypothalamic–Pituitary–Adrenal Axis

The gut microbiome has been suggested to play a role in the regulation of the HPA axis, potentially influencing stress responsivity and contributing to the development and modulation of limbic and hippocampal circuits. Microbial metabolites have been proposed to interact with hypothalamic neurons and glial cells, with experimental evidence suggesting potential modulation of corticotropin-releasing hormone (CRH), adrenocorticotropin (ACTH), and glucocorticoid levels, including cortisol. These effects are thought to occur through a combination of pathways, including vagal afferent signaling, systemic immune signaling via cytokines, and potential epigenetic regulation of gene expression; however, much of the evidence remains derived from preclinical models or indirect human data [62,63,64].

Early-life alterations in the gut microbiome have been associated in experimental and observational studies with changes in HPA axis regulation, which may manifest as altered stress responsivity, including both increased and decreased hormonal reactivity. Sustained elevations in cortisol have been linked in preclinical and clinical studies to changes in hippocampal, prefrontal, and amygdala structure and function, and have been associated with impairments in memory processes, synaptic plasticity, and emotional regulation, as well as with anxiety- and depressive-like phenotypes. However, these associations remain complex and do not establish direct causal pathways in humans. Conversely, reduced HPA axis reactivity has been proposed to impair adaptive stress responses and environmental sensitivity, potentially affecting cognitive and behavioral flexibility. Hormonal signaling may also exert local effects at synaptic and glial levels, where it has been suggested to contribute to circuit maturation and plasticity [62,65,66].

SCFAs have been shown in preclinical studies to exert epigenetic effects through modulation of DNA methylation and histone acetylation. These effects have been associated with regulation of genes involved in neuronal plasticity, excitability, and glucocorticoid receptor expression in brain regions such as the hypothalamus, hippocampus, and amygdala [67,68]. Microbial neurotransmitters may influence neuronal excitability, oscillatory activity, and excitatory–inhibitory balance within limbic circuits, while dopamine is known to play a key role in reward and motivation systems. Glial cells, including microglia and astrocytes, have been shown in preclinical models to respond to microbial-derived signals by participating in synaptic pruning and supporting dendritic maturation, thereby contributing to synaptic homeostasis and experience-dependent plasticity [69,70]. Although these mechanisms provide a biologically plausible framework linking the gut microbiome and stress regulation systems, their integration into a unified causal model of HPA axis dysregulation in neurodevelopmental and neuropsychiatric disorders remains speculative. Differences in stress responsivity across disorders may involve partially overlapping pathways, including SCFA-related epigenetic modulation and neurotransmitter signaling; however, the extent to which these represent shared causal mechanisms in humans has not yet been established.

### 3.4. Regulation of Neurotransmitters and Excitatory–Inhibitory Circuits

The gut microbiome has been suggested to influence the maturation of excitatory–inhibitory neuronal circuits through metabolic, enzymatic, and paracrine pathways, potentially affecting neurotransmitter precursor availability and postsynaptic receptor function. By metabolizing tryptophan and other essential amino acids, the microbiota may influence peripheral and central levels of serotonin and dopamine precursors, which have been associated in experimental and observational studies with serotonergic and dopaminergic signaling in brain regions such as the prefrontal cortex, hippocampus, and basal ganglia. These pathways have been proposed to be relevant for cognitive domains including attention, impulse control, motivation, and learning; however, direct causal evidence in humans remains limited [71,72]. Microbial production of neuroactive compounds such as GABA and glutamate has been shown in preclinical studies to influence neuronal excitability and excitatory–inhibitory balance, primarily through local effects on enteric neurons and glial cells, with potential downstream signaling pathways that may indirectly affect central nervous system function. Early-life microbiota alterations have been associated in experimental and observational studies with changes in cortical excitability states, including both hyperexcitability and hypoexcitability, which have been linked to altered network development and increased vulnerability to neurodevelopmental and neuropsychiatric conditions such as autism spectrum disorder, ADHD, and pediatric depression [73,74,75]. However, these associations remain complex and do not establish direct mechanistic causality in humans. The proposed shared molecular pathways may contribute to overlapping circuit-level vulnerabilities observed across ADHD, ASD, and related neurodevelopmental conditions, although the extent to which these mechanisms are causal or compensatory remains unresolved.

In addition, the gut microbiome has been proposed to interact with broader neuromodulatory systems, including neuropeptides and hormonal metabolites, which are known to regulate receptor systems such as NMDA, AMPA, and GABA-A. Experimental evidence suggests that these interactions may influence excitatory–inhibitory circuit stability, synaptic maturation, dendritic spine morphology, and neuronal oscillatory synchronization; however, much of this evidence is derived from preclinical models [76,77]. Overall, the gut microbiome is increasingly considered a potential contributor to neurochemical and neurodevelopmental regulation during early life, although its role should currently be interpreted as modulatory and context-dependent rather than as a primary causal driver of cognitive, behavioral, or emotional outcomes.

### 3.5. Modulation of the Systemic Immune Response and Neuroinflammation

The gut microbiome has been proposed to play a role in immunological homeostasis and neuroinflammatory regulation, with potential downstream effects on CNS development. By modulating the balance between proinflammatory cytokines (IL-1β, IL-6, TNF-α) and anti-inflammatory cytokines (IL-10, TGF-β), influencing macrophage polarization, and promoting regulatory T cell (Treg) activity, the microbiota may contribute to shaping systemic immune tone, which has been suggested to indirectly influence microglial and astrocyte function. These immune-mediated signals are thought to affect glial phenotypes and their capacity to support neuronal maturation, synaptic formation, and activity-dependent synaptic pruning. Most of this communication is considered to occur indirectly, via systemic immune, paracrine, and endocrine pathways, as well as through modulation of microglial metabolic states, rather than direct central penetration, thereby potentially influencing synaptic plasticity and excitatory–inhibitory circuit development [78,79].

Microbial metabolites, including SCFAs, tryptophan derivatives, and other secondary metabolites, have been proposed to act as metabolic and epigenetic modulators. Experimental evidence suggests that these signals may influence gene expression in neurons, microglia, and astrocytes, potentially through pathways such as NF-κB, MAPK, and mTOR, which are involved in cellular processes relevant to synaptogenesis, neuronal excitability, and excitatory–inhibitory balance [80,81]. Through these mechanisms, microbiota-derived signals have been associated in preclinical studies with modulation of NMDA, AMPA, and GABA-A receptor function, and may contribute to changes in dendritic spine density, dendritic arborization, and synaptic organization during development. Hyperactive or dysregulated microglial states, particularly in the context of persistent inflammation or early-life immune perturbation, have been linked in experimental and observational studies to alterations in cortical and limbic circuits involved in attention, emotional regulation, and cognitive processing [82]. Overall, these findings suggest that microbiota–immune–glia interactions may contribute to the modulation of neuroinflammatory responses and may be involved in neural circuit maturation. These processes are best interpreted as context-dependent and multi-level interactions that may influence excitatory–inhibitory network development, rather than as coordinated or deterministic mechanisms. Accordingly, potential associations have been suggested with aspects of cognitive development, emotional regulation, and behavioral outcomes; however, these relationships remain indirect and not yet causally established in humans [78,79,80,81,82].

### 3.6. Influence on the Integrity of the Blood–Brain Barrier

The gut microbiome has been suggested to play a role in the development and functional maintenance of the blood–brain barrier (BBB), primarily through microbial metabolites such as SCFAs, tryptophan derivatives, and phenolic compounds. These metabolites may modulate the expression of tight junction proteins (claudins, occludins, ZO-1), which are thought to be involved in regulating endothelial permeability, potentially via pathways including Wnt/β-catenin signaling and epigenetic mechanisms. SCFAs have also been proposed in preclinical studies to interact with pericytes and astrocytes, where they may influence the production of neurotrophic factors such as BDNF and GDNF, thereby contributing to the regulation of the neuronal microenvironment and potentially supporting the development of excitatory–inhibitory circuits [83,84,85,86].

Alterations such as intestinal dysbiosis, chronic systemic inflammation, or early-life microbiota imbalance have been associated with changes in BBB integrity, including reduced expression of tight junction proteins. These conditions have been suggested to promote a shift in microglial activity toward proinflammatory states; however, causality in humans remains incompletely established. Increased BBB permeability has been hypothesized to allow greater peripheral immune and metabolic signaling into the CNS, including cytokines, LPS, and other microbial-associated molecules, which may contribute to local immune activation and oxidative stress, particularly in cortical, hippocampal, and limbic regions. These processes have been associated in experimental and observational studies with alterations in dendritic architecture, synaptic density and plasticity, and neuronal network synchronization. In addition, microbiota–immune interactions may indirectly influence neuroimmune feedback systems, in which circulating cytokines and microbial metabolites are proposed to modulate microglial and astrocyte function, thereby contributing to local inflammatory signaling and homeostatic regulation within the CNS. These interactions are best interpreted as multi-level and context-dependent processes that may influence neuronal responsiveness to metabolic and immune cues, particularly during early development [83,87,88,89]. Overall, current evidence suggests that an appropriately balanced early-life microbiota may be associated with BBB integrity and neuroimmune signaling; however, its role in directly shaping neurocognitive outcomes remains indirect and not yet causally established.

### 3.7. Modulation of Social and Emotional Behavior

The gut microbiome has been proposed to influence the maturation of limbic and higher cortical circuits involved in social behavior, emotional regulation, and stress responses, with potential effects on the functional connectivity of regions such as the amygdala, hippocampus, and prefrontal cortex. However, current evidence is largely derived from preclinical and indirect human studies, and causal relationships in humans remain incompletely established. Microbial metabolites, particularly tryptophan derivatives and SCFAs, have been suggested to modulate gene expression pathways involved in synaptic plasticity, dendritic maturation, and synaptogenesis. These mechanisms have been associated in experimental models with processes relevant to the development of neural circuits implicated in social cognition, reward processing, and behavioral regulation; however, their direct relevance to human neurodevelopment requires further validation. The microbiome has also been proposed to interact with the HPA axis, limbic monoaminergic systems, and dopaminergic pathways within the nucleus accumbens, potentially influencing stress responsivity, emotional processing, social motivation, and impulse control. These effects are primarily supported by preclinical evidence and are not yet fully characterized in human developmental contexts. Early-life microbiota disturbances, such as dysbiosis or chronic systemic inflammation, have been associated with alterations in microglial function and excitatory–inhibitory balance, which may contribute to changes in neural circuit maturation. Such alterations have been linked in observational and experimental studies to behavioral phenotypes including anxiety-like behavior, social avoidance, altered stress reactivity, and repetitive behaviors; however, these associations do not establish direct causal pathways in humans. While phenotypic manifestations vary across disorders, SCFA- and neurotransmitter-related modulation of limbic and cortical circuits has been proposed as a potentially shared biological pathway underlying vulnerability in conditions such as ASD, ADHD, and anxiety-related disorders. However, this interpretation remains hypothesis-generating rather than mechanistically confirmed. Overall, the gut microbiome may contribute to the modulation of neural systems involved in social and emotional development during early life, but current evidence supports a modulatory and context-dependent role rather than a primary causal or integrative regulatory function in neurodevelopmental disorders [11,69,70].

### 3.8. Integration of Metabolic and Nutritional Signals

The gut microbiome has been proposed to function as a metabolic and nutritional contributor to host physiology, producing a range of bioactive metabolites, including SCFAs, essential fatty acids, microbial-derived vitamins (e.g., B complex vitamins and vitamin K), and amino acid-derived compounds, particularly those associated with the tryptophan–serotonin pathway. These metabolites have been suggested to influence central nervous system development and function through epigenetic, metabolic, and neurotrophic pathways [29,90,91,92]. However, most evidence supporting these effects is derived from preclinical or indirect human studies. These microbial-derived compounds have been associated in experimental models with changes in gene expression in neurons and glial cells, as well as with processes relevant to dendritic spine maturation, receptor expression (ionotropic and metabotropic), synaptogenesis, axonal myelination, neuronal energy metabolism, redox regulation, and neurotransmitter synthesis. These effects remain primarily mechanistic hypotheses based on preclinical data rather than established human pathways. Short-chain fatty acids, particularly butyrate, have been shown in experimental systems to act as histone deacetylase inhibitors, and have been suggested to facilitate epigenetic regulation processes that may be relevant for synaptic plasticity and network stabilization. Tryptophan metabolites have been associated with modulation of serotonin biosynthesis and aryl hydrocarbon receptor signaling in preclinical studies, with potential implications for neuroimmune regulation and microglial development [93,94]. Microbial-derived vitamins are thought to support enzymatic processes involved in neurotransmission, energy metabolism, and cellular proliferation, although their direct relevance to brain development in humans remains incompletely characterized [90]. Disruptions in microbial composition, dietary insufficiency, or intestinal inflammation have been associated with alterations in metabolic signaling pathways and may be linked to changes in cortical and limbic circuit development, synaptic efficiency, and dendritic architecture. These changes have been associated in observational and experimental studies with differences in cognitive domains such as executive function, attention, working memory, and information processing; however, causal relationships with pediatric neuropsychiatric disorders remain unproven [95]. Overall, current evidence supports a potential modulatory role of microbiota-derived metabolites in neurodevelopmental processes, although their contribution to neuropsychiatric susceptibility is best interpreted as indirect, multifactorial, and not yet causally established.

In conclusion, the gut microbiome has been proposed to contribute to neurodevelopment through a network of molecular, synaptic, and systemic interactions, mediated by metabolic, immunological, and hormonal signals that may influence microglial activity, synaptogenesis, myelination, and cortico-limbic circuit maturation. Early-life microbial imbalances have been associated with alterations in synaptic plasticity, excitatory–inhibitory balance, blood–brain barrier integrity, and HPA axis function; however, current evidence does not support a direct causal relationship with cognitive, emotional, or neuropsychiatric outcomes in humans. Overall, the microbiome may act as a modulatory component of brain development rather than a central regulatory system, with effects spanning cellular and circuit-level processes (summarized in Table 1). Despite the heterogeneity of pediatric neurodevelopmental disorders, SCFA-related signaling, neurotransmitter pathways, and microglial activity have been proposed as potentially shared biological mechanisms in preclinical and translational studies, although their unifying relevance in humans remains to be established. Maintaining microbiota and gut barrier homeostasis during early developmental windows has been suggested as potentially relevant for supporting normal neurodevelopment; however, evidence for specific nutritional, probiotic, or prebiotic interventions remains preliminary and requires further controlled longitudinal validation.

## 4. Implications in Psychiatric Disorders

Against the backdrop of increasing evidence of bidirectional gut–brain communication and the recognized role of the microbiota during early life, intestinal dysbiosis has been proposed as a factor of potential relevance to neurodevelopment. Disruption of microbial balance during critical periods of nervous system maturation has been associated with long-term functional changes in experimental and observational studies and may be linked to alterations in neuronal circuit organization, neuroimmune signaling, and behavioral phenotypes; however, causal relationships in humans remain incompletely established. Beyond the molecular and physiological mechanisms described above, integrating these findings into a clinical context requires careful consideration of their heterogeneity and limitations. Correlational evidence may provide a broader perspective on potential disease mechanisms and could inform future hypotheses for early intervention strategies targeting the microbiota; however, current data remain insufficient to support clinical application. Although shared molecular pathways involving SCFAs, neurotransmitter systems, and microglial activity have been proposed across pediatric neurodevelopmental disorders, these should be interpreted as general and non-specific biological processes rather than disorder-defining mechanisms. Evidence strength varies considerably between conditions, with autism spectrum disorder (ASD) being the most extensively studied, while attention deficit hyperactivity disorder (ADHD), Tourette syndrome, and other disorders are supported by smaller, less consistent, or conflicting datasets. Importantly, microbial alterations should not be interpreted as uniform signatures of disease, but rather as context-dependent and potentially modifiable biological features. In particular, variability across studies is not necessarily contradictory but may reflect differences in cohort composition, environmental exposures, dietary patterns, medication use, and methodological approaches such as sequencing depth, taxonomic resolution, and bioinformatic pipelines. Where available, inconsistencies and divergent findings are addressed within disorder-specific sections to better reflect the heterogeneity of the evidence base. Overall, current evidence across pediatric neurodevelopmental and neuropsychiatric disorders is heterogeneous, with limited reproducibility of specific microbial patterns. While ASD shows relatively more consistent findings, evidence for other conditions remains sparse or contradictory. Therefore, dysbiosis should be interpreted cautiously, and its role considered within disorder-specific and multifactorial frameworks rather than as a universal pathogenic mechanism.

### 4.1. Autism Spectrum Disorder

Autism spectrum disorder (ASD) is a complex neurodevelopmental condition characterized by persistent difficulties in social interaction, communication, and restricted or repetitive behaviors. In addition to the neurological phenotype, individuals with ASD frequently present with gastrointestinal symptoms, including constipation, abdominal pain, or discomfort, which has led to the hypothesis of a potential association between the intestinal microbiota and central nervous system function. This bidirectional communication, often referred to as the gut–microbiota–brain axis, involves multiple proposed mechanisms, including SCFA metabolism, immune modulation, and vagal signaling, through which the microbiota may indirectly influence neurodevelopment and behavior. Dysbiosis has been suggested in preclinical and observational studies to be associated with ASD-related phenotypes; however, causal relationships remain unconfirmed in humans and are limited by methodological differences across sequencing platforms and study designs [96].

Microbiota profiling studies in ASD have reported differences in microbial diversity and composition compared with neurotypical controls. Several studies describe alterations in taxa involved in fiber fermentation and SCFA production, including decreased abundance of *Prevotella* and *Bifidobacterium* (e.g., *B. breve*, *B. longum*), alongside variable changes in *Bacteroides*, *Faecalibacterium*, and *Clostridiales*-related taxa; however, these findings are highly dependent on sequencing resolution and may not reflect strain-level functional differences. Other studies report increased abundance of *Lachnospiraceae*, *Erysipelotrichaceae*, *Dorea*, and *Collinsella*, while overlapping or opposite patterns are also observed across cohorts, likely reflecting differences in diet, gastrointestinal comorbidities, and medication exposure. Systematic reviews have suggested shifts in Firmicutes and Bacteroidetes, although these are sensitive to analytical pipelines and should be interpreted cautiously. Certain taxa, including *Erysipelotrichaceae* and *Lachnospiraceae*, have been associated with symptom severity, and species such as Bifidobacterium longum and Streptococcus salivarius with milder social symptoms. Fungal alterations, including *Candida* and *Ascomycota*, have also been reported, although data remain largely cross-sectional, limiting causal inference. Enterotype-based and machine learning approaches have identified microbial patterns distinguishing ASD from controls; however, these findings remain exploratory and require external validation. Some studies suggest that milder ASD phenotypes show microbiota profiles closer to neurotypical individuals, although reproducibility remains limited, suggesting potential cohort-specific effects [97,98,99,100,101,102,103,104]. These factors likely introduce systematic bias, complicating the distinction between true biological signals and context-dependent variation.

Overall, inconsistencies across studies reflect a combination of biological and methodological factors, including host characteristics (age, severity, comorbidities), environmental influences (diet, medication exposure), and differences in sequencing and analytical pipelines. These sources of variation are difficult to fully control across studies and collectively limit direct comparability. Consequently, although recurrent microbial alterations are reported in ASD, no reproducible or disease-specific microbiota signature has been established. Current evidence supports an association between microbiota composition and ASD phenotypes, but its interpretation as a biomarker or therapeutic target remains preliminary and requires further longitudinal and mechanistic validation.

### 4.2. Childhood Depression

Major depression in children and adolescents is one of the most common mood disorders in this age group and is associated with significant emotional distress, functional impairment, and increased risk of suicide. Its prevalence appears to be rising globally, affecting up to 20% of adolescents. Early onset is a well-established predictor of adult depression and is linked to long-term impairments in social functioning, academic performance, and overall health. Risk factors include genetic vulnerability, early adverse experiences, chronic stress, sleep disturbances, and psychiatric comorbidities. Clinically, pediatric depression is characterized not only by affective symptoms but also by sleep and appetite disturbances, cognitive difficulties, and somatic complaints, which may complicate diagnosis. Although cognitive-behavioral therapy and pharmacological interventions remain first-line treatments, clinical response is heterogeneous and often incomplete [105,106,107,108].

Alterations in gut microbiota composition and function have been reported in children and adolescents with major depression. Multi-omics and 16S rRNA studies have described reduced abundance of SCFA-producing and putatively anti-inflammatory taxa, including *Faecalibacterium* and *Bifidobacterium animalis*, alongside increased representation of *Eggerthellaceae*, *Akkermansia*, and *Escherichia-Shigella*. Systematic reviews further report heterogeneous shifts in taxa involved in inflammation and amino acid metabolism, including *Alistipes*, *Parabacteroides*, *Flavonifractor*, *Oscillibacter*, *Streptococcus*, *Olsenella*, *Atopobium*, and *Bifidobacterium*, as well as reduced abundance of *Prevotella*, *Ruminococcaceae*, *Lachnospiraceae*, *Anaerostipes*, *Eubacterium*, *Dorea*, *Dialister*, and *Sutterella*. While these taxa are broadly linked to SCFA production and metabolic regulation, findings are not fully consistent across cohorts, likely reflecting differences in age, diet, disease severity, and medication exposure, as well as methodological variability in sequencing and taxonomic classification. At the functional level, alterations in microbial metabolic pathways—particularly those involved in SCFA production and amino acid metabolism—have been reported, predominantly involving members of the class *Clostridia*. However, most evidence remains associative, and the extent to which these changes directly contribute to central nervous system processes is still unclear. Early-life microbiota composition has also been associated with later internalizing symptoms, although these relationships are likely bidirectionally influenced by environmental and psychosocial factors such as early adversity, which itself may shape microbial composition and stress-related neurodevelopmental pathways [109,110,111,112].

Mechanistically, gut microbiota alterations in depression have been linked to tryptophan metabolism, including potential shifts toward the kynurenine pathway, as well as changes in SCFA availability. These pathways are hypothesized to interact with immune activation and neuroendocrine regulation, including the HPA axis, thereby influencing stress responsivity and mood regulation. However, most mechanistic evidence derives from preclinical or observational studies, limiting causal inference in humans. Adverse childhood experiences may further modulate these interactions by jointly affecting stress physiology and gut microbial composition, potentially increasing vulnerability to depressive phenotypes [113,114,115].

Overall, inter-study variability should be interpreted as reflecting combined biological and methodological heterogeneity rather than direct contradictions. Differences in cohort composition, environmental exposures, and analytical approaches contribute substantially to inconsistent microbial signals across studies. In particular, taxa such as *Bifidobacterium* show divergent directional changes across datasets, underscoring the absence of a stable, reproducible depression-specific microbiota signature. Consequently, current evidence supports an association between gut microbiota composition and pediatric depression phenotypes, but its use as a diagnostic biomarker or therapeutic target remains premature and requires further longitudinal and mechanistic validation.

### 4.3. Attention Deficit Hyperactivity Disorder

Attention deficit hyperactivity disorder (ADHD) is characterized by a persistent pattern of inattention, hyperactivity, and impulsivity that significantly interferes with a child’s social, academic, and family functioning. The global prevalence of ADHD in children and adolescents is estimated at approximately 4–7%, with variability depending on diagnostic criteria and study methodology, with some analyses reporting even higher values. ADHD typically begins in early childhood and may persist into adolescence and adulthood, with a proportion of cases showing long-term symptoms or functional impairment. Risk factors include a combination of genetic, neurobiological, perinatal, and environmental influences. The disorder is frequently accompanied by psychiatric comorbidities, including oppositional, conduct, anxiety, or mood disorders. ADHD symptomatology in children is often heterogeneous and may include difficulties in emotional regulation and social adaptation, highlighting the need for early multidisciplinary assessment and intervention [116,117].

Comparative analyses between children with ADHD and healthy controls have reported alterations in gut microbial diversity and variations in the relative abundance of certain bacterial species, particularly within the genera *Bacteroides* and *Sutterella*. In this context, increased abundance of *Bacteroides uniformis*, *Bacteroides ovatus*, and *Sutterella stercoricanis*, along with decreased abundance of *Bacteroides coprocola*, has been reported in association with ADHD, and in some cases with symptom severity, particularly for *B. ovatus* and *S. stercoricanis* [118]. Other studies describe reduced microbial α-diversity and decreased concentrations of SCFAs (including acetic, propionic, and valeric acid), which are metabolites considered relevant for immune and neurophysiological regulation [119]. Multi-omics studies in children with ADHD have reported a decrease in the phylum Actinobacteria and genera such as *Bifidobacterium*, *Corynebacterium*, and *Actinomyces*, alongside an increase in *Veillonella*, as well as correlations between *Bifidobacterium* abundance and precursor metabolites of neurotransmitters (dopamine, serotonin, glutamate), suggesting a possible association with neurochemical pathways, although causality has not been established [120]. Furthermore, a comparative study between children with ADHD and ASD reported that bacterial groups such as Clostridia, Ruminococcaceae, and Lachnospiraceae were more abundant in ADHD, while *Bacteroides*, *Bacilli*, and Actinobacteria were more frequent in ASD. In addition, probiotic supplementation increased microbial diversity only in ASD, without significant changes in ADHD [121]. A systematic review of 11 studies reported heterogeneous findings, including increased abundance of *Odoribacter* and *Eggerthella* and reduced levels of *Faecalibacterium prausnitzii* in some ADHD cohorts, with *F. prausnitzii* negatively correlated with symptom severity in certain studies, suggesting a potential association between microbial composition and neurochemical or immune-related processes [122]. Similar observations have been reported by Shirvani-Rad et al. [123]. Microbiota differences have also been associated with dietary patterns and nutrient intake, suggesting that diet may act as an important modulator of both microbial composition and metabolite production involved in gut–brain axis signaling. In addition, a longitudinal study reported that early-life microbiota composition (at 1 and 6 months of age), including bacterial and fungal taxa, was associated with later ADHD diagnosis at age 10, suggesting that early microbial differences may precede clinical manifestation, although these findings remain observational [124].

Importantly, the variability observed across ADHD microbiome studies should be interpreted as the result of interacting biological and methodological sources of heterogeneity rather than conflicting evidence per se, including age, clinical severity, psychiatric comorbidities, dietary variability, pharmacological exposure (particularly psychostimulants and antibiotics), and methodological differences in sequencing platforms and bioinformatic pipelines (e.g., 16S rRNA sequencing versus metagenomics), all of which can substantially influence microbial readouts. These confounding factors likely introduce systematic bias in reported taxa abundance, making it difficult to distinguish true disease-associated microbial shifts from context-dependent variability. Overall, these data suggest a complex interaction between diet, microbiota, and neurodevelopmental outcomes in ADHD, where microbial variation may reflect both disease-related and developmental influences rather than a stable disease-associated signal, highlighting the need for further longitudinal and interventional studies.

Functional studies suggest that the gut microbiome may be involved in modulating neuronal processes implicated in ADHD through metabolic activity. A pilot study integrating microbiota profiling and neuroimaging reported that bacterial genes associated with dopamine precursor synthesis were correlated with ventral striatum activation during reward anticipation, a neural process relevant to ADHD symptomatology [125]. Metagenomic analyses further suggest that gut dysbiosis may influence metabolic pathways related to neurotransmitter synthesis through host–microbiome interactions. In this context, *Bifidobacterium* spp. (e.g., *B. longum*, *B. breve*) have been suggested to be associated with dopamine and dopaminergic precursors, while *Lactobacillus*, *Streptococcus*, *Escherichia*, *Morganella*, *Klebsiella*, and *Hafnia* spp., together with intestinal enterochromaffin cells, have been implicated in serotonin production. *Bifidobacterium*, *Lactobacillus*, and *Escherichia* spp. have also been associated with GABA production in experimental studies. Although these neurotransmitters do not directly cross the BBB, intestinal microbiota may influence circulating tryptophan availability via *Lactobacillus*, *Bifidobacterium*, *Clostridium sporogenes*, and *Clostridium bartlettii*, with potential relevance for fronto-striato-limbic circuitry involved in ADHD [126].

Emerging evidence also suggests that microbial metabolites derived from aromatic amino acid metabolism (phenylalanine, tyrosine, L-DOPA) and interactions with catecholaminergic pathways may be associated with variability in executive functions, ADHD symptoms, and social behavior in pediatric populations [127]. Additional microbial metabolites, such as imidazole propionate, have been shown to cross the BBB and influence neuronal energy metabolism and neuroendocrine responses. SCFAs and vitamin B6 (pyridoxal phosphate) are also proposed to support neurotransmitter metabolism and neuronal function, with experimental studies suggesting that their imbalance may be associated with hyperactivity and repetitive behaviors [126]. Overall, these findings suggest a potential association between microbiome-related metabolic pathways, neurotransmitter systems, and ADHD-related brain function [125,127].

Consistent microbiota alterations have been reported in ASD, ADHD, and pediatric depression; however, no reproducible, disorder-specific microbial signature has been identified. Instead, findings converge on broad shifts in taxa involved in SCFA production and immune–metabolic regulation, including *Faecalibacterium*, *Bifidobacterium*, and members of Ruminococcaceae, alongside variable changes in *Bacteroides*, Lachnospiraceae, and Clostridia-related groups. Importantly, these apparent overlaps likely reflect shared functional microbiome pathways combined with substantial methodological and biological heterogeneity, rather than true convergent disease-specific microbial profiles. Differences in cohort composition, diet, medication exposure, and sequencing methodologies contribute significantly to inter-study variability and limit cross-condition comparability. Overall, current evidence supports a model of non-specific microbiota–brain interactions in pediatric neurodevelopmental and psychiatric disorders, where microbial changes represent context-dependent and multi-factorial associations rather than diagnostic or mechanistic signatures.

### 4.4. Rett Syndrome

Rett syndrome (RTT) is a severe, progressive, multisystemic genetic neurodevelopmental disorder, determined in over 95% of cases by de novo mutations in the *MECP2* gene, located on the X chromosome, which encodes the MeCP2 protein involved in the regulation of gene expression and normal neuronal function. Mutations in this gene lead, after an initial period of apparently typical development, to regression of motor and cognitive skills, loss of communication abilities, respiratory abnormalities, epilepsy, and other systemic manifestations. The clinical course progresses through distinct stages and requires multidisciplinary management. The global prevalence is estimated at approximately 1 in 10,000–20,000 live births, with no major ethnic differences reported. Prognosis varies depending on mutation severity and access to long-term supportive care. Preclinical studies have shown that restoration of *MECP2* expression in animal models can reverse neurological phenotypes, which has increased interest in disease-modifying approaches, including gene-based and pharmacological strategies targeting MeCP2-related pathways and neuronal circuit dysfunction [128,129,130].

Analyses of gut microbiota and fecal metabolome in girls and young women with RTT have shown that overall bacterial composition may not differ significantly from that of control groups, while functional metabolic profiles appear altered. In particular, reduced fecal levels of GABA, tyrosine, and glutamate have been reported, suggesting differences in microbial or host–microbiota metabolic interactions in RTT [131]. Within the same cohorts, microbiome variability has been associated with clinical severity, pubertal stage, and dietary patterns, indicating that microbial structure is strongly context-dependent and influenced by host developmental and environmental factors, which complicates interpretation of disease-specific effects. Metagenomic studies have also reported reduced microbial diversity and alterations in bacterial and fungal community structure in RTT. Reported changes include relative increases in genera such as *Bifidobacterium*, *Clostridium*, Erysipelotrichaceae, *Actinomyces*, *Lactobacillus*, *Enterococcus*, *Eggerthella*, *Escherichia/Shigella*, and *Candida*, alongside alterations in SCFA profiles and markers of low-grade intestinal inflammation, such as elevated fecal calprotectin and erythrocyte sedimentation rate. However, these findings are not consistently correlated with gastrointestinal symptoms such as constipation, suggesting that microbiome and metabolic changes may reflect a broader systemic phenotype rather than a direct gut-specific pathological mechanism [132]. This also limits causal interpretation, as cross-sectional designs preclude temporal inference between microbial variation and clinical features. Additional studies have reported reduced microbial diversity in RTT compared with healthy controls, along with functional changes in fecal metabolomic profiles. Relative differences in taxa such as Bacteroidaceae, *Clostridium*, *Sutterella* spp., and reductions in Ruminococcaceae have been described, together with variations in SCFAs, including butyrate and propionate, as well as branched-chain fatty acids. Functional predictive analyses suggest shifts in microbial genes involved in amino acid and SCFA metabolism, while carbohydrate and lipid metabolism pathways appear more represented in healthy controls. Dietary differences, including a higher relative protein intake in RTT patients, may also contribute to variability in microbial composition and metabolite production [133]. Importantly, heterogeneity in sequencing approaches, small cohort sizes, and variability in clinical stratification across studies further limit reproducibility and cross-study comparability of reported microbial patterns. Rather than indicating a uniform microbial disruption, the available evidence suggests that microbiome findings in RTT are highly context-sensitive, emerging at the intersection of disease stage, metabolic state, and external influences such as diet. This makes it challenging to disentangle whether observed microbial shifts are primary features of RTT biology or downstream reflections of systemic and behavioral constraints associated with the disorder. As a result, current evidence should be interpreted as associative rather than mechanistic or diagnostic. Taken together, these findings suggest an association between RTT and functional alterations of the gut microbiota–metabolome axis; however, available evidence remains limited in sample size and heterogeneous across studies. At present, no consistent or disease-specific microbial signature has been established for RTT, and the functional relevance of observed changes remains to be fully clarified.

### 4.5. Intellectual Developmental Disorders

Emerging evidence suggests that the gut microbiome may be associated with cognitive function and neuronal plasticity. In experimental models, the absence of microbiota in germ-free mice has been shown to induce transcriptomic alterations in microglia within the prefrontal cortex and hippocampus, affecting genes involved in synaptogenesis, neurotransmission, and GABA metabolism. Recolonization with microbiota has been reported to partially restore some of these gene expression changes. These findings suggest a potential role of the microbiota in regulating neurodevelopmental processes; however, their direct translation to human cognitive phenotypes remains limited and requires further validation [134], particularly given differences in brain maturation trajectories and microbiota stability between animal models and humans, which constrain translational inference. Extension of these findings to clinical and experimental human contexts suggests that gut microbiota composition, diversity, and early colonization patterns may be associated with behavioral and cognitive outcomes. Reported microbial alterations have been linked not only to neurotransmitter signaling and microglial activity, but also to immuno-metabolic and endocrine pathways that are thought to contribute to neural circuit development, as well as to BBB permeability and availability of neuroactive metabolites. While these associations support a potential role of gut dysbiosis in cognitive outcomes, causal relationships remain unproven. Accordingly, microbiota modulation has been proposed as a potential research avenue in intellectual developmental disorders, although clinical evidence remains preliminary and largely derived from small, heterogeneous, and methodologically diverse studies [135,136,137].

Clinical and experimental studies in humans have reported that reduced gut microbiota diversity or compositional imbalance is associated with lower cognitive performance, including impairments in working memory and attention, as well as symptoms of anxiety and depression. Some interventional studies have reported improvements in cognitive or adaptive outcomes following administration of probiotics or other microbiota-targeted interventions; however, these findings are variable and require further confirmation, as most studies are limited by short follow-up periods, small sample sizes, and lack of standardized cognitive outcome measures, which reduces reproducibility across cohorts [138]. Preclinical studies in murine models support the involvement of the microbiota in cognitive and behavioral processes, showing that alterations in microbial composition—such as increased abundance of *Bacteroidetes* and *Lactobacillaceae*, alongside decreased abundance of *Firmicutes*, *Proteobacteria*, *Actinobacteria*, and *Ruminococcaceae*—are associated with differences in cognitive performance and adaptive behavior. These findings provide experimental evidence of microbiota–brain interactions, although translational relevance to human cognition remains to be established, as interspecies differences in immune development, diet, and microbial ecology may substantially alter host–microbiome interactions [139]. In humans, some studies have reported associations between specific microbial taxa, such as *Bacteroides*, *Odoribacter*, and *Butyricimonas*, and better cognitive performance or structural features of the hippocampus, a brain region involved in memory and learning. These associations have been observed primarily in adult populations and do not directly reflect pediatric intellectual developmental disorders; however, they suggest a potential link between microbiota composition and cognitive-related brain structure and function, although these findings are constrained by cross-sectional designs, limiting inference regarding temporal directionality or causality [140].

Finally, emerging evidence in preschool children provides preliminary insight into early-life associations between microbiota and cognition. Reduced microbial diversity and differences in the abundance of taxa such as *Acinetobacter*, *Blautia*, *Faecalibacterium*, *Prevotella_9*, *Subdoligranulum*, *Collinsella*, *Dialister*, *Holdemanella*, and *Methanobrevibacter* have been associated with lower IQ scores, while certain fecal metabolites have been correlated with cognitive performance. These findings suggest that early-life microbial and metabolic profiles may be associated with cognitive development; however, causality and mechanistic pathways remain unclear, and are further complicated by confounding factors such as diet, antibiotic exposure, and socio-environmental variability during early development [141].

Overall, the current body of evidence points toward a biologically plausible relationship between the gut microbiome and cognitive function, but this relationship appears to be context-dependent and likely influenced by developmental stage and host–environment interactions rather than reflecting a stable, disease-specific microbial signature.

### 4.6. Childhood Epilepsy

Recent data suggest that the gut microbiota may be associated with modulation of epileptic seizure severity in infants, although the direction of causality remains unclear and cannot be inferred from current observational designs. In children with refractory epilepsy, the composition of the microbiota differs from that of healthy infants, being characterized by reduced microbial diversity and predominance of Proteobacteria, while Bacteroidetes and Actinobacteria are less represented. Nutritional interventions, such as the ketogenic diet, are associated with significant changes in the microbiota, with increases in Bacteroidetes and *Prevotella* and decreases in Proteobacteria and Firmicutes; notably, it remains unclear whether these microbial shifts directly mediate seizure reduction or reflect secondary consequences of systemic metabolic reprogramming induced by ketosis, as most available studies are cross-sectional or pre-post in design without mechanistic mediation analysis. However, the ketogenic diet is also associated with reductions in certain bacteria considered beneficial, such as *Bifidobacterium*, *Eubacterium rectale*, and *Dialister*, while *Escherichia coli* may increase, suggesting a functional and metabolic remodeling of the microbiota. Recent preclinical data indicate that the composition of ketogenic formulations, particularly dietary fiber content, may influence microbiota structure and seizure susceptibility in murine models, with fiber intake being associated with a microbial profile linked to increased seizure resistance. Clinical response to the ketogenic diet has been reported to correlate with specific microbial species and circulating metabolites, with plasmalogens being associated with reduced seizure frequency and with the presence of bacteria such as *Faecalibacterium prausnitzii*, *Alistipes communis*, *Alistipes shahii*, and *Christensenella minuta*, while strains of *Escherichia coli* and certain infant-type *Bifidobacterium* show negative correlations. Patients who do not respond clinically to the ketogenic diet often exhibit higher abundances of microbial groups such as *Clostridiales*, *Ruminococcaceae*, *Rikenellaceae*, *Lachnospiraceae*, and *Alistipes*, suggesting that specific microbial configurations may be associated with variability in therapeutic response. These observations indicate that intestinal microbial balance, influenced by both dietary composition and host metabolic state, is associated with seizure susceptibility and response to dietary therapy; however, causality and mechanistic directionality remain to be fully established [142,143,144,145,146]. Overall, current evidence supports a bidirectional and highly complex interaction between diet, microbiota, and host metabolism, rather than a unidirectional microbiota-driven mechanism, with current findings being largely associative and derived from heterogeneous study designs that differ in population characteristics, dietary protocols, sequencing depth, and analytical pipelines.

Importantly, these associations should be interpreted within a broader framework of biological and methodological heterogeneity, as differences in cohort composition, clinical severity, microbiome profiling methods, and dietary adherence likely contribute substantially to variability in reported taxa and metabolic signatures across studies. In addition, variability in reported microbial taxa and metabolite profiles across studies further limits the identification of a consistent epilepsy-associated microbiota signature and constrains cross-study reproducibility.

### 4.7. Spectrum of Tourette Syndrome and Tic Disorders

Although the etiopathogenic mechanisms of tic spectrum disorders remain incompletely elucidated, recent literature indicates a possible role of the gut microbiome in modulating brain development and function. Current reviews suggest that gut dysbiosis may contribute to neurodevelopmental vulnerability through mechanisms involving systemic and neuroinflammation, microglial activation, altered immunological responses, and regulation of neurotransmitters such as dopamine and GABA. Alterations in the gut–brain axis may be associated with neuronal excitability and the function of cortico-striatal-thalamic circuits, potentially facilitating the emergence or worsening of tics, although a causal relationship cannot be established based on current evidence, particularly given that most available data derive from observational and cross-sectional designs with limited longitudinal validation [147,148].

In this regard, available data indicate the existence of specific variations in the composition of the intestinal microbiota in patients with tic spectrum disorders compared to healthy subjects. The studies analyzed reported, among others, an increased relative abundance of taxa such as *Ruminococcaceae* and *Bacteroides*, as well as changes at the species level, including *Bacteroides plebeius* and *Ruminococcus lactaris*. In parallel, reductions in bacteria such as *Prevotella stercorea*, *Streptococcus lutetiensis*, and *Bifidobacterium* have been described, suggesting a microbial imbalance with potential functional implications. Increases in genera such as *Faecalibacterium*, *Hungatella*, *Oscillibacter*, *Flavonifractor*, *Fusicatenibacter*, *Anaerostipes*, *Anaerotruncus*, and *Eisenbergiella* have also been identified in children with Tourette syndrome, indicating an altered microbial profile potentially associated with intestinal pro-inflammatory and metabolic changes. Of particular interest are observations regarding taxa involved in neurotransmitter metabolism, especially GABA, where the abundance of species associated with GABA production, such as *Bifidobacterium* spp., *Eubacterium* spp., and *Akkermansia muciniphila*, has been reported to be inversely correlated with tic severity, while bacteria involved in GABA degradation, such as *Klebsiella pneumoniae*, have been associated with clinical changes in symptom severity [148,149,150]. However, these findings are based on relatively small and heterogeneous cohorts, and genus-level resolution in 16S rRNA datasets may not accurately reflect functionally relevant strain-level differences.

Additional data indicate that certain genera, including *Agathobacter*, *Dorea*, *Anaerostipes*, *Butyricicoccus*, and *Bifidobacterium*, may change following combined physiotherapy interventions, correlating with improvements in tic severity, suggesting a potential bidirectional association between the gut microbiota and clinical phenotype in Tourette syndrome [150].

In addition, a recent meta-analysis confirmed that patients with tic disorders exhibit differences in microbial community structure (beta diversity) compared to healthy controls, while findings regarding alpha diversity remain inconsistent. Consistent changes in specific taxa were also reported, including increases in *Peptostreptococcaceae* and reductions in *Escherichia/Shigella* and *Roseburia*, suggesting that intestinal dysbiosis may act as a modulatory factor in tic-related neurodevelopment and behavior [151]. Nonetheless, cross-study heterogeneity in sequencing platforms, bioinformatic pipelines, and cohort composition significantly limits comparability, and may partially account for divergent findings across studies. These results should be interpreted cautiously, as cross-study differences in populations, methods, and analytical pipelines likely influence the microbial patterns reported and limit their direct comparability.

In support of these clinical observations, murine models indicate that experimental modulation of the gut microbiota, including fecal microbiota transplantation (FMT) or probiotic administration, is associated with changes in tic-like behaviors, alongside alterations in microbial composition and peripheral serotonin levels [152]. Recent clinical trial data in children with Tourette syndrome further show that FMT is associated with changes in gut microbiota composition, including increases in *Bacteroides coprocola* and *Dialister succinatiphilus* and changes in *Bacteroides vulgatus*, which were correlated with reductions in tic severity as measured by the Yale Global Tic Severity Scale in most participants [153]. However, these interventional findings remain preliminary and are limited by small sample sizes, short follow-up durations, and variability in donor and protocol standardization.

Overall, these findings support the hypothesis that intestinal dysbiosis may contribute to dysfunction in inhibitory neurotransmission and cortico-striato-thalamic circuit regulation, which are central in tic spectrum disorders; however, a causal link remains unproven due to limitations in current evidence [147,148,149,150,151,152,153].

However, reported microbial alterations vary considerably across studies, and findings regarding specific taxa are not consistently replicated. This heterogeneity, together with small sample sizes and methodological differences, limits the ability to define a clear microbiota profile associated with tic disorders.

### 4.8. Motor and Language Development Disorders

There is emerging evidence suggesting that the early gut microbiota may be associated with neurodevelopmental trajectories, including motor and language acquisition, although data remain limited and largely derived from small longitudinal cohorts with substantial inter-individual variability. Longitudinal studies indicate that variations in microbial composition in infants may correlate with patterns of functional brain connectivity and aspects of behavior, which in turn may be associated with motor and cognitive development. In particular, taxa such as *Clostridiales* and members of the *Lachnospiraceae* family appear to be associated with differences in neural networks involved in emotion regulation and motor control, suggesting an indirect association between the microbiome and neurocognitive development. These observations support the hypothesis that microbial diversity and the presence of specific bacteria in the first months of life may contribute to the maturation of brain circuits involved in movement and language, indicating a potential mechanism through which early dysbiosis may influence developmental outcomes. However, evidence of causality is lacking, and further longitudinal studies integrating metagenomic profiling with neuropsychomotor assessments are needed, particularly studies with standardized sampling time points and harmonized analytical pipelines to reduce inter-study variability [112,154].

Moreover, evidence suggests that the effects of early microbiota are not limited to emotional networks but may also involve fronto-parietal and sensorimotor circuits implicated in motor control and language acquisition. Taxa such as *Bifidobacterium*, *Bacteroides*, *Prevotella*, *Roseburia*, *Eubacteriaceae*, *Anaerobutyricum*, *Dialister*, and *Veillonella* have been identified in several studies as being associated with differential patterns of brain connectivity and with emergent temperaments or behaviors that may influence motor and language development. These associations suggest the existence of early “microbial signatures” that may be associated with the maturation of neural circuits involved in motor and cognitive regulation. However, most data derive from observational or cross-sectional studies, and causal relationships remain unconfirmed, with additional uncertainty introduced by heterogeneity in cohort characteristics, nutritional exposures, and microbiome profiling methods [155].

In addition, recent evidence indicates that certain early microbial configurations may be associated with specific aspects of language development and cognitive functions. For example, increased abundance of *Bacteroidetes* at one year of age was associated with higher scores on cognition and language tests at two years, particularly in boys, suggesting that early colonization with these microbes may be associated with maturation of neural circuits involved in language processing and cognitive functions [156]. Similarly, another study identified that distinct microbial structures in one-year-old infants, including groups with higher alpha diversity and the presence of specific taxa, correlate with cognitive and language scores at two years, highlighting that variations in early microbiota composition may be associated with neurocognitive development, including expressive language [157]. However, these associations are sensitive to confounding factors such as diet, breastfeeding status, antibiotic exposure, and socioeconomic environment, which are not consistently controlled across studies.

Furthermore, microbial profiles in 18-month-old infants, including the presence of genera such as *Turicibacter* and *Parabacteroides* associated with lower fine motor scores, and *Collinsella*, *Coprococcus*, *Enterococcus*, *Fusobacterium*, *Holdemanella*, *Propionibacterium*, *Roseburia*, *Veillonella*, *Bifidobacterium*, and *Lactobacillus* associated with higher scores, were correlated with fine motor development, suggesting that early microbiota may be associated with both cognitive and motor components of infant development [158]. This observation complements previously identified microbial signatures, highlighting the importance not only of microbial diversity but also of the presence of specific taxa that may be associated with neurocognitive and language development. Nevertheless, functional interpretation of these taxa remains limited, as most studies rely on taxonomic associations without direct metabolomic or mechanistic validation.

However, the precise mechanisms remain unclear, and further studies, preferably longitudinal and integrated with metagenomic profiling and detailed neuropsychological assessments, are needed to further clarify how early dysbiosis may be related to language development and cognition. Findings across studies remain based largely on observational designs, and differences in population characteristics, developmental timing of sampling, and analytical approaches likely influence reported associations, limiting direct comparability between studies. As such, conclusions regarding causality and specific microbial signatures should be considered preliminary.

Overall, while specific microbiota alterations vary across neurodevelopmental disorders, converging evidence suggests the involvement of shared molecular pathways, including SCFA-mediated signaling, neurotransmitter modulation, and microglial activity, in shaping neurodevelopmental outcomes. Although these shared pathways suggest a common biological framework, their downstream effects likely depend on developmental timing, genetic background, and environmental exposures, which may help explain the disorder-specific phenotypes observed. However, most available data remain associative, with significant heterogeneity across studies, and causal relationships have not been established.

Future research integrating longitudinal clinical data with mechanistic approaches will be essential to clarify these interactions and to identify potential targets for early microbiota-based interventions. Considering all of the above, Table 2 summarizes the key findings of microbial alterations across neurodevelopmental disorders. Given the heterogeneity of study designs, this table provides a structured overview of the main evidence. To further enhance clarity and accessibility of the evidence base, a more detailed synthesis of all included studies is provided in Supplementary Table S1. This supplementary table extends Table 2 by systematically organizing individual studies according to design, population/model, taxa reported, and main outcomes. It is intended as a qualitative synthesis rather than a quantitative comparison, given the heterogeneity of study designs and the inclusion of both clinical and preclinical evidence. The directionality of changes (increase/decrease) reflects dominant trends reported across studies rather than uniform findings. Both human clinical studies and preclinical experimental models were included when they provided mechanistic insight into gut–brain interactions; however, differences in translational validity are acknowledged, and findings from animal studies should be interpreted as hypothesis-generating rather than clinically confirmatory.

## 5. Therapeutic Perspectives

The prevalence of neurodevelopmental and mental health disorders in the pediatric population constitutes a major challenge for health systems, affecting approximately 15% of children and adolescents. These conditions, including affective, anxiety and obsessive–compulsive disorders, autism spectrum disorders and ADHD, are associated with a significant and lasting impact on functioning and clinical outcome. Given their chronic nature, current research aims to expand conventional therapeutic approaches by integrating pharmacological and complementary interventions. In this context, the gut–brain axis, especially the role of diet quality and gastrointestinal dysfunction as modifiable factors, has become a major area of interest, offering new perspectives for personalized therapeutic interventions [159,160].

Interventions targeting the gut microbiota are emerging and still largely experimental strategies for modulating neurodevelopment and symptoms associated with pediatric neuropsychiatric disorders, including autism spectrum disorder (ASD), ADHD, RTT, and other conditions frequently accompanied by gastrointestinal comorbidities. Probiotics, such as *Lactobacillus strains* (e.g., *Lactobacillus reuteri*), administered prenatally or early in life, have been reported in small, heterogeneous, and predominantly exploratory studies to be associated with reductions in ASD-related behaviors and improvements in gastrointestinal symptoms. However, these findings remain preliminary, non-confirmatory, and limited by small sample sizes, short follow-up periods, and lack of standardized outcome measures. Any suggested longer-term neurodevelopmental effects (e.g., ADHD- or Asperger-like manifestations) should therefore be interpreted strictly as hypothesis-generating, given the absence of robust longitudinal evidence. Proposed mechanisms, including modulation of microbial composition, metabolic output, and immune signaling, are largely derived from preclinical or indirect evidence and do not establish clinical efficacy in humans. Accordingly, current data support a mechanistic and exploratory role rather than evidence of therapeutic effectiveness in pediatric populations. Fecal microbiota transplantation (FMT) may contribute to transient restoration of microbial diversity; however, evidence in children with ASD is currently limited to small, uncontrolled, or variably designed studies with inconsistent outcomes. Reported improvements in gastrointestinal or behavioral domains should therefore be interpreted cautiously, as they are constrained by methodological limitations, small sample sizes, and insufficient control for confounding factors. Importantly, FMT remains an experimental approach in pediatric populations, and its use raises relevant safety concerns, including potential transmission of infectious agents, antimicrobial resistance genes, and unknown long-term metabolic and immunological consequences in the developing host. Early nutrition, including intake of prebiotic oligosaccharides from breast milk and diets rich in fiber and anti-inflammatory nutrients, may support beneficial microbial colonization, SCFA production, and intestinal barrier integrity. However, evidence linking these mechanisms to measurable neurodevelopmental outcomes in humans remains limited, indirect, and largely associative [161,162].

In pediatric populations—particularly in children with ASD, ADHD, or restrictive eating patterns such as ARFID—dietary behaviors represent major and often insufficiently controlled confounders in microbiota–brain axis research. Selective or restrictive eating patterns, commonly characterized by low fiber intake and high consumption of ultra-processed foods, are independently associated with alterations in gut microbiota composition and metabolic activity. Evidence from human studies indicates that dietary intake can rapidly and reproducibly modify the gut microbiome, highlighting diet as a key upstream determinant of microbial variation [163]. In ASD, food selectivity and restrictive eating behaviors are highly prevalent and may significantly influence both nutritional intake and microbiota profiles, independent of neurodevelopmental status [164,165]. Consequently, it remains difficult to determine whether observed microbial differences are causally involved in neurodevelopmental phenotypes or primarily reflect underlying dietary and behavioral patterns. While the gut–diet–brain axis represents a complex bidirectional system, current evidence does not support a defined causal direction in pediatric neurodevelopmental disorders [36]. Therefore, available data do not justify firm conclusions regarding a direct causal role of gut microbiota in ASD or ADHD, particularly given the predominance of cross-sectional designs and limited dietary control. Accordingly, microbiota-targeted interventions should currently be regarded as exploratory, adjunctive, and non-causative strategies, with observed effects potentially influenced by unmeasured dietary and lifestyle confounders.

Recent systematic reviews and meta-analyses suggest that microbiota-targeted interventions, including probiotics and other microbiome-modulating strategies, may exert modest and context-dependent effects. However, these findings remain preliminary, heterogeneous, and not yet sufficient to support clinical conclusions. Reported changes in microbial diversity or composition should not be directly interpreted as clinical benefit. Although some studies report that probiotic supplementation (e.g., 12-week interventions) may increase microbial diversity in children with ASD or modify specific bacterial taxa in ADHD populations, the clinical relevance of these findings remains uncertain and non-confirmatory. Similarly, while FMT and multi-strain probiotic formulations have been associated in some studies with improvements in behavioral outcomes, particularly social functioning, these effects are inconsistent across cohorts and not reproducible in a robust manner. Descriptions of strain-specific effects (e.g., *Lactobacillus rhamnosus* GG, *Bifidobacterium longum*) should therefore not be interpreted as evidence of targeted efficacy, due to lack of comparative and mechanistic validation in pediatric clinical settings. Overall, proposed mechanisms—including immune modulation, neuroactive metabolite production, and gut–brain axis signaling—remain largely hypothetical in human pediatric studies [121,166,167,168,169].

Interventions targeting the gut microbiota in Rett syndrome (RTT) and tic disorders, including Tourette syndrome, remain exploratory and are supported by limited evidence. In RTT, probiotics and synbiotics may improve gastrointestinal symptoms, although evidence for behavioral effects remains weak and inconsistent. FMT has been proposed only as an extrapolated strategy, without direct clinical validation in RTT [162,170]. In tic disorders, preliminary data suggest that microbiota modulation may be associated with changes in tic severity; however, current evidence is insufficient to establish efficacy, reproducibility, or clinical relevance [149]. Overall, microbiota-targeted interventions across RTT, Tourette syndrome, ASD, and ADHD should be considered experimental, with uncertain efficacy and context-dependent safety profiles [149,162,170].

Although microbiota-targeted interventions are increasingly investigated, current evidence remains limited by small sample sizes, heterogeneous methodologies, variable intervention protocols, and inconsistent outcome measures. These limitations are present across all intervention types discussed. Additional uncertainties include optimal dosage, timing of intervention, strain specificity, and long-term effects. FMT, in particular, raises unresolved concerns regarding donor variability, standardization, and long-term safety in pediatric populations, including microbiome stability and systemic metabolic effects. Importantly, all current findings should be interpreted within the context of exploratory and non-confirmatory evidence. While early microbiota alterations may have potential predictive value for neurodevelopmental outcomes, existing data are insufficient to support causal or clinically actionable conclusions. Overall, these limitations underscore the need for well-controlled, randomized, longitudinal, and mechanistic studies to clarify both efficacy and safety in pediatric populations [171].

Despite growing evidence supporting gut–brain interactions, current findings across studies remain inconsistent. Variability in study design, microbial profiling methods (e.g., 16S rRNA vs. shotgun metagenomics), population heterogeneity, and confounding factors contribute substantially to this inconsistency. Many proposed mechanisms are derived from preclinical models and do not consistently translate to human pediatric populations, highlighting a persistent translational gap. Accordingly, this review adopts an integrative narrative synthesis rather than a comparative or meta-analytic framework, aiming to identify recurring directional trends rather than definitive effect estimates. This approach avoids artificial precision that could arise from heterogeneous datasets. At present, microbial alterations appear context-dependent and vary across age, diet, medication exposure, and disease severity. Therefore, findings are presented within disorder-specific contexts and integrated at a functional level only. Overall, current evidence remains preliminary, heterogeneous, and insufficient to support causal or clinically definitive conclusions regarding microbiota-targeted interventions in pediatric neurodevelopmental disorders.

## 6. Conclusions

Neurodevelopment is a highly dynamic and interconnected process, with the gut microbiome serving as a pivotal regulator of the gut–brain axis. Evidence indicates that the microbiota shapes CNS maturation through neuroimmune, neuroendocrine, and metabolic mechanisms, influencing synaptogenesis, microglial development, excitatory–inhibitory balance, stress response, and BBB integrity. These processes are particularly sensitive during the perinatal period and early childhood, when plasticity is maximal. Dysbiosis during these critical windows can disrupt neuronal circuit organization and neuroimmune programming, increasing susceptibility to neurodevelopmental and neuropsychiatric disorders such as autism, ADHD, anxiety–depressive disorders, RTT, and tic disorders. While causal relationships remain incompletely defined, clinical and experimental data converge to support microbial imbalance as a risk factor and modulator of disease severity, rather than a sole determinant. Microbiota-targeted strategies—including optimized early nutrition, prebiotics, probiotics, synbiotics, and FMT in carefully selected cases—show promise as complementary approaches. Meta-analyses suggest modest but meaningful benefits, particularly in ADHD and specific behavioral domains of autism, with favorable safety profiles, though heterogeneity, small sample sizes, and lack of standardization limit current applicability. Future research should focus on longitudinal studies, identification of microbial biomarkers, optimal intervention windows, and personalized approaches. Integrating the gut microbiome into early biological programming could transform microbiota modulation into an effective preventive and therapeutic tool, with a lasting impact on children’s neurocognitive and emotional health.

## Figures and Tables

**Table 1:** The influence of the gut microbiome on central nervous system development.

Level/Component	Cellular Targets	Functional Processes	Communication Pathways	Molecules/Signals	Disturbances	Consequences/Final Effects
Microglia/CNS	Microglia	Maturation, phagocytosis, synaptic pruning	Metabolic, immunological, endocrine (cytokine-mediated) signaling	SCFAs, GABA, dopamine, cytokines	Early-life dysbiosis, maternal immune activation, systemic inflammation, genetic susceptibility, stress exposure	Impaired synaptic pruning, altered myelination, increased neurodevelopmental vulnerability
Synapses/neuronal plasticity	Neurons, astroglia	Synaptogenesis, dendritic remodeling, plasticity	Neuron–glia crosstalk, epigenetic regulation, neurotrophin signaling	SCFAs, BDNF, NGF, cytokines, microbial metabolites	SCFA deficiency, oxidative stress, nutritional imbalance, early inflammatory priming	Altered synaptic density, unstable dendritic spines, impaired learning and cognition
HPA axis	Hypothalamus, pituitary, adrenal glands	Stress response regulation, glucocorticoid secretion	Vagal signaling, cytokines, endocrine feedback loops	CRH, ACTH, cortisol, SCFAs, serotonin precursors	Early-life stress, dysbiosis, chronic inflammation, altered caregiving environment	HPA axis dysregulation, limbic dysfunction, impaired stress adaptation
Excitatory–inhibitory circuit	Cortical & limbic neurons	Network excitability balance, oscillatory activity	Metabolic and neurotransmitter-mediated signaling	GABA, glutamate, serotonin, dopamine, SCFAs	Neurotransmitter imbalance, microbial dysbiosis, developmental metabolic stress	Cortical hyper/hypoexcitability, ASD/ADHD-like phenotypes, cognitive dysregulation
Neuroimmune/neuroinflammation	Microglia, astrocytes, Tregs	Immune homeostasis, inflammatory tone regulation	Cytokine signaling, endocrine–immune crosstalk	IL-1β, IL-6, TNF-α, IL-10, TGF-β, SCFAs, tryptophan metabolites	Chronic low-grade inflammation, immune activation, gut barrier dysfunction	Aberrant microglial activation, synaptic dysfunction, neurodevelopmental vulnerability
Blood–brain barrier (BBB)	Endothelial cells, pericytes, astrocytes	Barrier integrity, permeability regulation	Wnt/β-catenin signaling, metabolic and epigenetic control	SCFAs, tryptophan derivatives, microbial phenolics	Dysbiosis, systemic inflammation, metabolic stress, oxidative damage	Increased permeability, neurotoxin entry (e.g., LPS), circuit dysfunction
Social & emotional behavior	Amygdala, hippocampus, PFC	Limbic circuit maturation, socio-emotional regulation	HPA axis, monoaminergic signaling, microbial metabolites	SCFAs, serotonin, dopamine, GABA	Early-life dysbiosis, stress exposure, inflammatory priming	Anxiety, social withdrawal, repetitive behaviors, emotional dysregulation
Metabolic & nutritional integration	Neurons, glia	Energy metabolism, synaptogenesis, myelination	Metabolic, hormonal, epigenetic signaling	SCFAs, vitamins (B, K), amino acids, tryptophan metabolites	Malnutrition, dysbiosis, intestinal inflammation	Reduced plasticity, impaired executive function, altered neurodevelopment

Note: The described mechanisms may manifest differently across pediatric disorders; disorder-specific effects are influenced by host genetics, environmental factors, and microbiome composition.

**Table 2:** Altered gut bacterial profiles in neurodevelopmental disorders and their functional relevance.

Pathology	Affected Microbial Profiles	Observed Changes	Important Notes
Autism spectrum disorders	*Prevotella* †, *Bifidobacterium* (*B. breve*, *B. longum*) †, *Bacteroides* †, *Faecalibacterium* †, *Oscillospira*, *Clostridium* †, *Lachnospiraceae*, *Clostridiales*, *Erysipelotrichaceae*, *Dorea*, *Collinsella*, *Corynebacterium*, *Lachnoclostridium*, *Parasutterella*, *Paraprevotella*, *Alistipes*, *Bilophila*, *Dialister*, *Veillonella*, *Streptococcus salivarius; Candida*, *Ascomycota*, *Firmicutes*, *Bacteroidetes*	**Decreases**: *Prevotella* †, *Bifidobacterium* †, *Parasutterella*, *Paraprevotella*, *Alistipes*, *Bilophila*, *Dialister*, *Veillonella*, (in some studies: *Bacteroides* †, *Faecalibacterium* †) **Increases**: *Oscillospira*, *Clostridium* †, *Lachnospiraceae*, *Clostridiales*, *Erysipelotrichaceae*, *Dorea*, *Collinsella*, *Corynebacterium*, *Lachnoclostridium*, *Firmicutes*, *Candida*, *Ascomycota*, (in some studies: *Bacteroides* †, *Faecalibacterium* †)	Imbalances (*B. longum* and *Strepto-coccus salivarius*) correlate with symptom severity and behavioral phenotype; there are distinct “microbial signatures” and enterotypes associated with severity.
Childhood depression	*Faecalibacterium* †, *Bifidobacterium animalis* †, *Roseburia*, *Eggerthellaceae*, *Akkermansia*, *Escherichia-Shigella*, *Alistipes*, *Parabacteroides*, *Flavonifractor*, *Oscillibacter*, *Streptococcus*, *Olsenella*, *Atopobium*, *Dorea*, *Dialister*, *Sutterella*, *Prevotella*, *Anaerostipes*, *Eubacterium*, *Ruminococcaceae*, *Lachnospiraceae*	**Decreases**: *Faecalibacterium* †, *Roseburia*, *Bifidobacterium animalis* †, *Prevotella*, *Ruminococcaceae*, *Lachnospiraceae*, *Anaerostipes*, *Eubacterium*, *Dorea*, *Dialister*, *Sutterella* **Increases**: *Eggerthellaceae*, *Akkermansia*, *Escherichia-Shigella*, *Alistipes*, *Parabacteroides*, *Flavonifractor*, *Oscillibacter*, *Streptococcus*, *Olsenella*, *Atopobium*, (in some studies: *Bifidobacterium* †)	Reduced SCFA and increased pro-inflammatory taxa disrupt tryptophan metabolism and neurotransmission; early microbiota may “program” neural networks involved in emotion regulation.
Attention Deficit Hyperactivity Disorder	*Bacteroides* (*B. uniformis*, *B. ovatus*, *B. coprocola*) †, *Sutterella* (*S. stercoricanis*) †, *Actinomyces*, *Corynebacterium*, *Veillonella*, *Clostridia*, *Ruminococcaceae*, *Lachnospiraceae*, *Odoribacter*, *Eggerthella*, *Bifidobacterium* †, *Lactobacillus*, *Streptococcus*, *Escherichia*, *Morganella*, *Klebsiella*, *Hafnia*, *Clostridium* spp., *Faecalibacterium* †, *Faecalibacterium prausnitzii* †, *Actinobacteria*	**Decreases***: B. coprocola* †, *α-diversity*, *Bifidobacterium* †, *Corynebacterium*, *Actinomyces*, *Faecalibacterium* †, *F. prausnitzii* †, *Actinobacteria* **Increases**: *B. uniformis* †, *B. ovatus* †, *S. stercoricanis* †, *Veillonella*, *Clostridia*, *Ruminococcaceae*, *Lachnospiraceae*, *Odoribacter*, *Eggerthella*, *Lactobacillus*, *Streptococcus*, *Escherichia*, *Morganella*, *Klebsiella*, *Hafnia*, *Clostridium* spp.	Microbial changes may influence neurotransmitter production (dopamine, serotonin, GABA); microbial metabolites and vitamin B6 contribute to brain function; early microbial profile (1–6 months) may precede ADHD. Evidence remains heterogeneous.
Rett syndrome	*Bifidobacterium*, *Clostridia*, *Erysipelotrichaceae*, *Actinomyces*, *Lactobacillus*, *Enterococcus*, *Eggerthella*, *Escherichia/Shigella*, *Candida*, *Bacteroidaceae*, *Sutterella* spp., *Ruminococcaceae*	**Decreases**: *Ruminococcaceae* **Increases**: *Bifidobacterium*, *Clostridia*, *Erysipelotrichaceae*, *Actinomyces*, *Lactobacillus*, *Enterococcus*, *Eggerthella*, *Escherichia/Shigella*, *Candida*, *Bacteroidaceae*, *Sutterella*	Functional dysbiosis, reduced diversity, and metabolic alterations (GABA, glutamate, tyrosine); link to severity of gastrointestinal and neurological phenotype.
Intellectual developmental disorders	*Bacteroidetes*, *Lactobacillaceae*, *Firmicutes*, *Proteobacteria*, *Actinobacteria*, *Ruminococcaceae*, *Acinetobacter*, *Blautia*, *Faecalibacterium*, *Prevotella_9*, *Subdoligranulum*, *Collinsella*, *Dialister*, *Holdemanella*, *Methanobrevibacter*, *Odoribacter*, *Butyricimonas*	**Decreases**: *Firmicutes*, *Proteobacteria*, *Actinobacteria*, *Ruminococcaceae*, *Acinetobacter*, *Blautia*, *Faecalibacterium*, *Prevotella_9*, *Subdoligranulum*, *Collinsella*, *Dialister*, *Holdemanella*, *Methanobrevibacter* **Increases:***Bacteroidetes*, *Lactobacillaceae*	Reduced diversity and microbial imbalances are associated with lower cognitive performance; specific fecal metabolites correlated with cognitive performance; evidence remains heterogeneous.
Childhood epilepsy (baseline microbiota)	*Proteobacteria*, *Bacteroidetes*, *Actinobacteria*	**Decreases**: *Bacteroidetes*, *Actinobacteria* **Increases**: *Proteobacteria*	Reduced diversity; microbiota alterations associated with seizure susceptibility.
Childhood epilepsy (ketogenic diet-induced changes)	*Prevotella*, *Escherichia coli*, *Bifidobacterium*, *Eubacterium rectale*, *Dialister*, *Firmicutes*	**Decreases**: *Proteobacteria*, *Firmicutes*, *Bifidobacterium*, *Eubacterium rectale*, *Dialister* **Increases***: Bacteroidetes*, *Prevotella*, *Escherichia coli*	Represents intervention-induced changes (diet), not baseline disease profile; may influence treatment response.
Tourette syndrome and tic disorders	*Ruminococcaceae*, *Bacteroides* †, *Prevotella stercorea*, *Streptococcus lutetiensis*, *Bifidobacterium*, *Faecalibacterium* †, *Hungatella*, *Oscillibacter*, *Flavonifractor*, *Fusicatenibacter*, *Anaerostipes*, *Anaerotruncus*, *Eisenbergiella*, *Escherichia/Shigella*, *Peptostreptococcaceae*, *Dialister*, *Eubacterium*, *Akkermansia muciniphila*, *Klebsiella pneumoniae*	**Decreases**: *Prevotella stercorea*, *Streptococcus lutetiensis*, *Bifidobacterium*, *Escherichia/Shigella*, *Roseburia* **Increases***: Ruminococcaceae*, *Bacteroides* †, *Faecalibacterium* †, *Hungatella*, *Oscillibacter*, *Flavonifractor*, *Fusicatenibacter*, *Anaerostipes*, *Anaerotruncus*, *Eisenbergiella*, *Peptostreptococcaceae*, *Dialister*	Imbalances affect GABA metabolism; microbial changes correlate with symptom severity and can be modulated by treatments and fecal transplantation; findings are heterogeneous across studies.
Motor and language development disorders	*Clostridiales*, *Lachnospiraceae*, *Bifidobacterium*, *Bacteroides*, *Prevotella*, *Roseburia*, *Eubacteriaceae*, *Anaerobutyricum*, *Dialister*, *Veillonella*, *Turicibacter*, *Parabacteroides*, *Collinsella*, *Coprococcus*, *Enterococcus*, *Fusobacterium*, *Holdemanella*, *Propionibacterium*, *Lactobacillus*, *Bacteroidetes*	**Decreases** (negative associations): *Turicibacter*, *Parabacteroides Variable/unclear direction: Bacteroides*, *Prevotella*, *Dialister*, *Anaerobutyricum* **Increases** (positively associated): *Bifidobacterium*, *Lactobacillus*, *Collinsella*, *Coprococcus*, *Enterococcus*, *Fusobacterium*, *Holdemanella*, *Propionibacterium*, *Roseburia*, *Veillonella*, *Bacteroidetes*	Data are largely observational; early “microbial signatures” associated with both cognitive and motor development; the data comes largely from observational studies, causality not established.

† Indicates taxa with inconsistent or contradictory findings across studies.

## Data Availability

No new data were created or analyzed in this study. Data sharing is not applicable to this article.

## Supplementary Materials

The following supporting information can be downloaded at: https://www.mdpi.com/article/10.3390/nu18101541/s1, Table S1: Overview of studies reporting gut microbiota alterations across neurodevelopmental and psychiatric disorders, including study design, population/model, taxa reported, and main findings.
