# Supplementary material for: Early Gut Microbiota and Neurodevelopmental Trajectories: Implications for Pediatric Neuropsychiatric Vulnerability—A Narrative Review
Source: Nutrients. 2026 May 13;18(10):1541. doi: 10.3390/nu18101541 (PMC13209934; doi:10.3390/nu18101541)
Supplement: Supplementary file 1 [file nutrients-18-01541-s001.zip › nutrients-4244019-supplementary.pdf]

Supplementary Table S1: Overview of studies reporting gut microbiota alterations across neurodevelopmental and psychiatric disorders, including study design, population/model, taxa reported, and main findings.

Ref	Study	Domain	Evidence Type	Population/Model	Taxa Reported	Key Findings (Direction + Outcome)
96	Lewandowska-Pietruszka 2025	Autism spectrum disorder	Human observational (cross-sectional)	Children	Bacteroides, Bifidobacterium, Firmicutes, Faecalibacterium	↓ Beneficial bacteria → ↑ ASD severity, gastrointestinal dysfunction
97	Chen 2021	Autism spectrum disorder	Human observational (case-control)	Children	Bacteroides, Prevotella, Bifidobacterium	Dysbiosis → social impairment, reduced cognitive performance
98	Ding 2020	Autism spectrum disorder	Human observational (case-control)	Children	Firmicutes, Bacteroidetes	Altered ratio → ASD diagnosis association
99	Zeng 2025	Autism spectrum disorder	Human observational (cross-sectional)	Children	Faecalibacterium, Bifidobacterium	↓ Faecalibacterium → symptom severity ↑
100	Strati 2017	Autism spectrum disorder	Human observational (case-control)	Children	Clostridium spp., Bacteroides	Overgrowth of Clostridium → gastrointestinal + behavioral symptoms
101	Jung 2024	Autism spectrum disorder	Human observational (cross-sectional)	Children	Bacteroides, Ruminococcaceae	Microbial ecotypes → symptom heterogeneity
102	Chen 2025	Autism spectrum disorder	Human observational (case-control family study)	Children + relatives	Bacteroides, Prevotella	Shared microbial patterns → familial microbiome signature
103	Lewandowska-Pietruszka 2023	Autism spectrum disorder	Systematic review	Human studies	Multiple taxa	Consistent dysbiosis across ASD studies
104	Peralta-Marzal 2024	Autism spectrum disorder	Human multi-cohort machine learning study	Multi-cohort	Microbial signature (multi-taxa)	Predictive microbiome profile for ASD classification
105	Liu 2025	Depression	Systematic review	Adolescents	Not uniformly reported	Gut–brain axis dysfunction in depression
106	Lu 2024	Depression	Meta-analysis	Population studies	Not applicable	Epidemiology of depression
107	Selph 2019	Depression	Clinical guideline review	Children/adolescents	Not applicable	Clinical management framework
108	Pataki 2016	Depression	Narrative review	Pediatric population	Not applicable	Clinical features of MDD
109	Teng 2025	Depression	Human observational (case-control)	Children/adolescents	Faecalibacterium, Roseburia, Bifidobacterium	↓ SCFA-producing bacteria → depressive symptoms ↑
110	Bai 2026	Depression	Human observational (cohort)	Adolescents	Akkermansia, Alistipes	ACE-linked dysbiosis → depression severity ↑
111	Cao 2026	Depression	Narrative review	Human studies	Multiple taxa	Dysbiosis → neurotransmitter + inflammation pathways
112	Querdasi 2025	Internalizing symptoms	Human longitudinal cohort	Children	Bacteroides, Firmicutes	Early microbiome → brain connectivity → symptoms
113	Zhou 2023	Depression	Human observational (case-control)	Adolescents	Bifidobacterium, Lactobacillus	Tryptophan pathway disruption → mood alteration
114	Cheng 2025	Depression	Human observational (case-control)	Adolescents	Streptococcus, Escherichia–Shigella	Inflammatory taxa ↑ → depressive symptoms
115	Bai 2024	Depression	Narrative review	Human studies	Multiple taxa	ACE → microbiome-mediated mood effects
116	Popit 2024	ADHD	Meta-analysis	Population studies	Not applicable	ADHD prevalence
117	Njardvik 2025	ADHD	Systematic review	Pediatric population	Not applicable	Psychiatric comorbidities
118	Wang 2020	ADHD	Human observational (case-control)	Children	Bacteroides, Sutterella	Dysbiosis → ADHD severity
119	Steckler 2024	ADHD	Human observational (case-control)	Children	Faecalibacterium, Ruminococcaceae	↓ SCFA bacteria → cognitive dysfunction
120	Han 2025	ADHD	Human observational (case-control)	Children	Bacteroides, Clostridia	Microbial imbalance → ADHD symptoms
121	Novau-Ferré 2025	ADHD/ASD	Human interventional (randomized controlled trial)	Children	Bifidobacterium, Lactobacillus	Probiotics → symptom improvement
122	Gkougka 2022	ADHD	Systematic review	Human studies	Multiple taxa	Consistent dysbiosis patterns
123	Shirvani-Rad 2022	ADHD	Systematic review	Human studies	Multiple taxa	Gut–brain axis involvement
124	Cassidy-Bushrow 2023	ADHD	Human longitudinal cohort	Children	Bacteroides, Firmicutes	Early microbiome → ADHD risk
125	Aarts 2017	ADHD	Human observational (cross-sectional)	Adolescents	Bifidobacterium	Reward system modulation
126	Boonchooduang 2020	ADHD	Narrative review	Human studies	Not specified	Gut–brain axis hypothesis
127	Krajewski 2025	ADHD	Human observational	Children	Multiple taxa	Metabolite-linked behavioral regulation
128	Ali 2025	Rett syndrome	Narrative review	Not applicable	Not applicable	MECP2 dysfunction
129	Lopes 2024	Rett syndrome	Narrative review	Not applicable	Not applicable	Clinical trials
130	Vashi 2019	Rett syndrome	Narrative review	Not applicable	Not applicable	Therapeutic development
131	Thapa 2021	Rett syndrome	Human observational (case-control)	Patients	Bifidobacterium, Clostridia	Dysbiosis → GI + neurological severity
132	Strati 2016	Rett syndrome	Human observational (case-control)	Patients	Bacteroidaceae	Reduced diversity → severity
133	Borghi 2017	Rett syndrome	Narrative review	Human studies	Multiple taxa	Microbiome involvement
134	Huang 2023	Neuroinflammation	Animal experimental	Mouse model	Microglia-associated taxa	Microbiome modulates neuroinflammation
135	Al Noman 2025	Neuroplasticity	Narrative review	Human studies	Not specified	Microbes influence brain plasticity
136	Warner 2019	Neurodevelopment	Narrative review	Human studies	Not specified	Gut–brain axis role
137	Zhu 2020	Neuroinflammation	Narrative review	Human studies	Multiple taxa	Inflammatory pathways
138	Cooke 2022	Cognition	Systematic review	Human studies	Not specified	Cognition–microbiome link
139	Putri 2023	Cognition	Animal meta-analysis	Rodents	Multiple taxa	Behavioral modulation
140	Liang 2022	Cognition	Human multi-omics	Mixed	Bacteroides, Faecalibacterium	Brain structure associations
141	Long 2024	Cognition	Human observational	Children	Faecalibacterium	IQ associations
142	Xie 2017	Epilepsy	Human interventional	Infants	Bacteroidetes, Firmicutes	Ketogenic diet → seizure reduction
143	Zhang 2018	Epilepsy	Human interventional	Children	Firmicutes, Bacteroidetes	Seizure improvement
144	Lindefeldt 2019	Epilepsy	Human interventional	Children	Multiple taxa	Diet-induced microbiome change
145	Dahlin 2024	Epilepsy	Human observational	Children	Multiple taxa	Metabolite–seizure link
146	Özcan 2025	Epilepsy	Animal experimental	Mouse model	Bacteroides, Firmicutes	Seizure resistance ↑
147	Borrego-Ruiz 2024	Neurodevelopmental disorders	Narrative review	Human studies	Multiple taxa	Dysbiosis across disorders
148	Wu 2025	Tourette syndrome	Narrative review	Human studies	Not specified	Neuroinflammation
149	Geng 2023	Tourette syndrome	Narrative review	Human studies	Not specified	Microbiome involvement

150	Bao 2023	Tourette syndrome	Human interventional (pilot)	Children	Bacteroides, Ruminococcaceae	Symptom reduction
151	Yang 2025	Neurodevelopmental disorders	Meta-analysis	Human studies	Multiple taxa	Cross-disorder dysbiosis
152	Li 2022	Tourette syndrome	Animal experimental	Mouse model	Lactobacillus, Bacteroides	Behavioral improvement
153	Zhao 2020	Tourette syndrome	Human interventional (pilot)	Children	Multiple taxa	Fecal transplantation effect
112	Querdasi 2025	Internalizing symptoms	Human longitudinal cohort	Children	Bacteroides, Firmicutes	Brain connectivity link
154	Kelsey 2021	Neurodevelopment	Human longitudinal cohort	Infants	Bacteroides-dominant	Temperament association
155	McMath 2023	Cognition	Systematic review	Human studies	Not specified	Cognitive development link
156	Tamana 2021	Neurodevelopment	Human cohort	Infants	Bacteroides-dominant	Neurodevelopment outcome
157	Carlson 2018	Cognition	Human longitudinal cohort	Infants	Diversity profiles	Cognitive scores
158	Acuña 2021	Motor development	Human observational	Infants	Bifidobacterium, Lactobacillus	Motor skill development
